# Theoretical design and analysis of D–π–A–A′ organic dyes for enhanced efficiency in DSSCs by modifying donor (D) and acceptor (A) moieties

**DOI:** 10.1039/d5ra07258a

**Published:** 2025-11-03

**Authors:** Smiti Rani Bora, Banashmita Barman, Dhruba Jyoti Kalita

**Affiliations:** a Department of Chemistry, Gauhati University Guwahati-781014 India dhrubajyoti.kalita@gauhati.ac.in

## Abstract

This study presents a comprehensive investigation of ten different dyes using DFT and TD-DFT calculations. The dyes feature a D–π–A–A′ architecture with a thiophene π-bridge, cyanoacrylic acid serving as both an electron-acceptor and anchoring group, and a selection of electron-donor and electron-acceptor moieties. The electron-donor moieties explored include coumarin (COU), triphenylamine (TPA), indoline (IN), carbazole (CAR), diphenylamine (DPA), tetrahydroquinoline (THQ), triazatruxene (TAT), azulene (AZ), and julolidine (JUD). Besides, the electron-acceptor moieties consist of quinoline (QN), [1,2,5]thiadiazole[3,4-*c*]pyridine (PY), phthalimide (PTM), benzothiadiazole (BTZ), naphthalenediimide (NDI), benzothiazole (BZ), and pyridoquinazolinone (PYQ). The dyes are labeled with the following designations: COU–QN, TPA–PY, IN–PTM, CAR–BTZ, DPA–NDI, THQ–BZ, TAT–BZ, TPA–PYQ, AZ–QN, and JUD–BTZ. Among these dyes, TPA–PYQ shows the lowest *Δ*_H–L_ value of 1.674 eV, which decreases further to 1.281 eV upon binding with the Ti_5_O_10_ cluster. Band-alignment plots indicate that all dyes have GSOP values below the redox potential of the I^−^/I_3_^−^ electrolyte (*i.e.*, −4.85 eV), while their ESOP values are generally above the TiO_2_ conduction band (*i.e.*, −4.05 eV), with the exception of AZ–QN. The negative adsorption energies suggest effective chemisorption of the dye–clusters on the TiO_2_ surface, facilitating electron transfer from the dye's LUMO to the conduction band of TiO_2_. Additionally, absorption studies reveal that the *λ*_max_ of the dyes shifts towards the red region when complexed with Ti_5_O_10_. Dye–clusters such as JUD–BTZ–Ti_5_O_10_, TPA–PY–Ti_5_O_10_, and TPA–PYQ–Ti_5_O_10_, with lower *E*_b_ values, exhibit enhanced exciton dissociation and charge transfer, leading to improved performance. These findings suggest that the designed dyes may act as promising candidates for the development of dye-sensitized solar cells (DSSCs).

## Abstract 2

This study presents a comprehensive investigation of ten different dyes using DFT and TD-DFT calculations.

## Graphical abstract

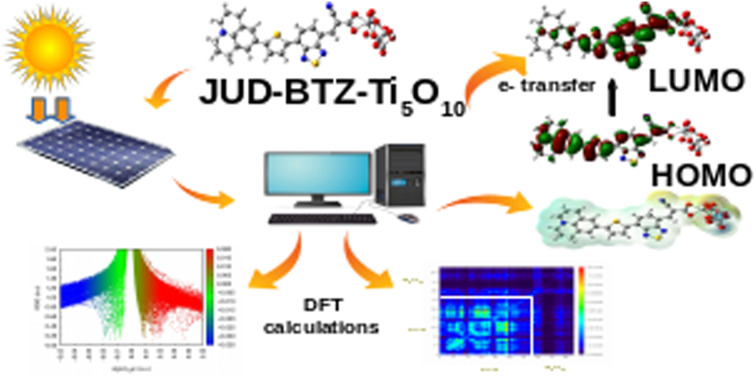

## 1 Introduction

Global energy consumption has surged by virtue of unprecedented population growth and technical advancements in industrial sectors driven by developmental initiatives. Scientists are encouraged to seek novel, sustainable, cost effective, and clean energy sources to meet global energy demands while addressing environmental concerns.^1^ Three generations of diverse technologies have been created with the purpose of capturing solar energy. Initially, crystalline silicon was utilized, followed by thin-film technologies, and now, the third generation employs organic materials, exemplified by dye-sensitized solar cells (DSSCs). Michael Grätzel and coworkers make substantial contributions to the academic and commercial research communities in 1991 with the development of DSSCs, a subset of thin-film solar cells.^2^ DSSCs are favored by scientists for their low toxicity, affordability, ease of production, and eco-friendliness. Understanding the fundamental principles and developmental strategies of DSSCs is crucial for achieving enhanced energy efficiency and long-term operational stability. Researchers are particularly interested in modifying DSSCs to improve their power conversion efficiencies (PCEs).^4^ Recent studies have shown promising results, with metal-free organic dye-based DSSCs achieving an impressive energy conversion efficiency of 15.2%.^5^

The key component within DSSC is the photosensitizing dye, which plays a vital role in determining the range of light absorption and the efficiency of energy harvesting.^3^ To be suitable for DSSCs, a dye must meet specific criteria, including high photostability at elevated temperatures, low production costs, effective adsorption on semi-conductive surfaces *via* anchoring groups, non-toxicity, a wide visible absorption spectrum, and a sufficiently high redox potential for dye regeneration after excitation.^6^ Metal-free organic dyes, typically fabricated with a D–A architecture, is mostly easy to design and modify, particularly for adjusting the energy levels of the highest occupied molecular orbital (HOMO) and lowest unoccupied molecular orbital (LUMO). Within the realm of conjugated organic materials, donor–π–acceptor (D–π–A) molecules represent a primitive class, where donor and acceptor units are linked with a π-conjugated bridge.^7–10^ Manipulation of the donor and acceptor units enable the modification of its physical and chemical properties. Molecule designed with the D–π–A architecture has garnered significant interest due to its versatile applications in molecular electronics, including organic light-emitting diodes (OLEDs), solar cell design, electrogenerated chemiluminescence, biochemical fluorescence technology, and the development of efficient nonlinear optical (NLO) materials. This architectural framework proves particularly advantageous in these mentioned areas, underscoring its significance in the advancement of electronic and optoelectronic technologies.^11^

In recent times, researchers have directed significant interest towards metal-free organic dyes due to their enhanced molar coefficients, minimal toxicity, ease of synthesis, and versatility.^12^ Typically, the essential mechanism for DSSC operation involves intramolecular charge transfer (ICT) from the donor to the acceptor upon light excitation. The design and molecular structure of organic dyes are pivotal factors in the fabrication of DSSCs. The primary goal is to enhance the PCE of DSSCs by altering the structure of the organic dye, aiming to develop devices that are affordable and can be synthesized easily, thus making them more suitable for broader use.^5^ Park *et al.* detailed a study on dyes for DSSCs utilizing a D–π–A–π–A architecture. These dyes incorporated various donor groups (–MeO, –MeS, and –Me_2_N) and acceptor units (benzothiadiazole and cyanoacrylic acid). Their research yielded a PCE of 5.61% and enhanced intramolecular charge transfer characteristics.^13^ Besides, Jadav *et al.* focused on TiO_2_ based DSSCs and explored the photovoltaic properties of four substituted coumarin dyes (MC1–MC4). They observed that dyes containing an electron withdrawing cyanogroup achieved a maximum efficiency of 4.60%, whereas those with only a hydrogen group attached to the dye exhibited an efficiency of 2.64%.^14^ Further, Marlina *et al.* explored ten organic dyes, all featuring a D–π–A–A architecture, incorporating two distinct auxiliary acceptors and five organic-based anchoring groups. They showed that the photovoltaic properties can be better tuned by varying the internal acceptor and anchoring group using density functional theory (DFT) and time-dependent DFT (TD-DFT) methods.^15^ Recently, DSSCs fabricated from dyes based on D–π–A–A architecture have been studied worldwide.^16–18^ It has been reported that incorporation of a double acceptor moiety into the dye structure can broaden the absorption band as well.^16^

**Fig. 1:**
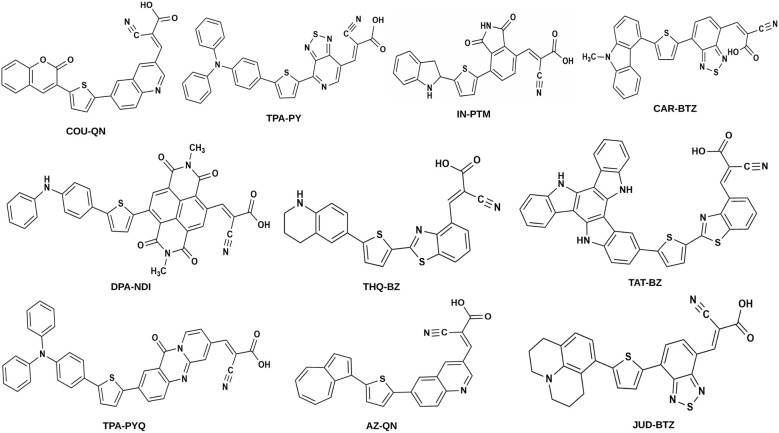
Sketches of the designed dyes.

In this work, we have designed a series of ten D–π–A–A′ dyes aimed at enhancing the performance of DSSCs. These ten dyes have been designed following the work of Han *et al.*^19^ As far as we know, there has not been much exploration into theoretical research on D–π–A–A′ dyes by varying the donor and acceptor units. These dyes incorporate various electron-donor (D) moieties such as coumarin (COU),^20^ triphenylamine (TPA),^21^ indoline (IN),^22^ carbazole (CAR), diphenylamine (DPA),^23^ tetrahydroquinoline (THQ),^24^ triazatruxene (TAT),^25^ azulene (AZ),^4^ and julolidine (JUD).^26^ Additionally, we have utilized electron-acceptor (A) moieties including quinoline (QN),^27^ [1,2,5]thiadiazole[3,4-*c*]pyridine (PY),^28^ phthalimide (PTM),^29^ benzothiadiazole (BTZ),^5^ naphthalenediimide (NDI),^30^ benzothiazole (BZ),^31^ and pyridoquinazolinone (PYQ).^32^ The dyes are designated as follows: COU–QN, TPA–PY, IN–PTM, CAR–BTZ, DPA–NDI, THQ–BZ, TAT–BZ, TPA–PYQ, AZ–QN, and JUD–BTZ. Furthermore, thiophene and cyanoacrylic acid serve as the common π-bridging unit and anchoring group (A′), respectively, for all designed dyes. Notably, carboxylic acids, such as cyanoacrylic acid, are commonly used for binding to TiO_2_ surfaces as electron acceptors.^5^ Moreover, we have employed Ti_5_O_10_ clusters as the semiconductor surface of TiO_2_. Sketches illustrating the designed dyes are provided in Fig. 1. The coordinates of the designed dyes are provided in Table S1 (in the SI). This study seeks to build a clear structure–property relationship for D–π–A–A′ dyes by systematically varying both the donor and acceptor units. The underlying idea is that an appropriate balance between donor strength and the acceptor's electron-withdrawing nature can simultaneously reduce the band gap, enhance light absorption, and promote efficient charge transport.

The ten dyes were chosen to systematically examine how varying donor and acceptor strengths influence the optoelectronic behavior of D–π–A–A′ systems. Each donor–acceptor pair was selected to represent a gradual variation in electron-donating and withdrawing capacities, allowing us to establish a clear structure–property relationship relevant to dye-sensitized solar cells.

## 2 Computational details

All calculations were conducted utilizing the Gaussian 09 program package.^33,34^ In order to validate the functionals employed, an extensive study was undertaken to enhance the accuracy of the results. A test calculation was performed employing a ZXY-3 dye, previously reported in literature for its structural similarity to our designed dyes.^19^ The optimized structure of the test compound is depicted in Fig. S1 (in the SI). Both ground and excited state calculations were performed using six different functionals, *viz.*, B3LYP/6-31G(d,p), B3LYP-D3/6-31G(d,p), CAM-B3LYP/6-31G(d,p), B3PW91/6-31G(d,p), HSEH1PBE/6-31G(d,p) and wB97XD/6-31G(d,p), employing density functional theory (DFT) and time-dependent DFT methods, respectively. We have correlated the calculated energies of HOMO and LUMO, difference between the energies of HOMO and LUMO (*Δ*_H–L_) and maximum absorption wavelength (*λ*_max_) values with experimental results. The results are reported in Table S2 (in the SI). Analysis of this table reveals that, for ground state calculations, the values obtained using the B3LYP-D3/6-31G(d,p) level of theory agree well with the experimental values. Conversely, for excited state calculations, the results obtained using the CAM-B3LYP/6-31G(d,p) level of theory exhibit better agreement with the experimental values. Therefore, ground and excited state calculations were performed at the B3LYP-D3/6-31G(d,p) and CAM-B3LYP/6-31G(d,p) levels of theory, respectively. Additionally, the M06-2X-D3/6-31G(d,p) level of theory was employed to compute the electronic coupling matrix element (*V*) values, as it incorporates dispersion correction and facilitates exploration of charge transfer properties.^35^ To study the absorption properties, 30 lowest-energy excitations (S_0_ → S_*n*_) were computed using TD-DFT. Besides, the optimization of dye–TiO_2_ clusters were conducted employing the B3LYP-D3 functional along with LANL2DZ basis set.

## 3 Theoretical methodology

The dihedral angle (*Φ*) indicates the angle between repeating units in π-conjugated systems, significantly affecting the molecule's planarity and polymer conjugation, which in turn influences the optoelectronic properties of the dyes. It also impacts the reorganization energy (*λ*) during hole/electron transport.^36,37^

The energy band gap, *Δ*_H–L_, is the energy difference between the HOMO and the LUMO. A lower *Δ*_H–L_ enhances the excitation efficiency of organic dyes and indicates greater stability. Eqn (1) and (2) are used to calculate the ionization potentials (IPs) and electron affinities (EAs) of the dyes:

1 IP = *E*^+^(*M*^o^) − *E*^o^(*M*^o^),

2 EA = *E*^o^(*M*^o^) − *E*^−^(*M*^o^).

The energies of the dyes in their cationic, neutral, and anionic states are represented as *E*^+^, *E*^o^, and *E*^−^, with the neutral geometry denoted as *M*^o^.

The reorganization energy (*λ*) measures the energy change due to structural reorganization of a dye molecule in response to excess charge. It has two main components: the outer sphere, involving electron relaxation or medium polarization, and the inner sphere, related to geometric changes from charge transfer. This study focuses on *λ* values from the inner sphere, calculated for cationic (*λ*_+_) and anionic species (*λ*_−_) using eqn (3) and (4).^38,39^

3 *λ*_+_ = [*E*^+^(*M*^o^) − *E*^o^(*M*^o^)] − [*E*^+^(*M*^+^) − *E*^o^(*M*^+^)],

4 *λ*_−_ = [*E*^o^(*M*^−^) − *E*^−^(*M*^−^)] − [*E*^o^(*M*^o^) − *E*^−^(*M*^o^)].

In this context, *M*^+^ and *M*^−^ represent the geometries of the dyes in their cationic and anionic forms, respectively.

A photovoltaic device's energy conversion efficiency (*η*) is typically expressed as:5
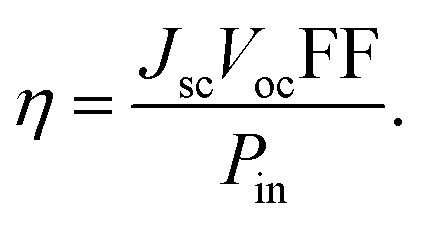
At this point, *J*_sc_, *V*_oc_, FF, and *P*_in_ represent the short-circuit photocurrent density, open-circuit voltage, fill factor, and input power from sunlight, respectively.

*J*_sc_ is defined as:6
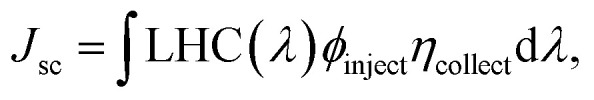
The measures of electron injection and collection are represented by *ϕ*_inject_ and *η*_collect_, respectively.

Two key parameters for calculating the dye's efficiency are its light harvesting capacity (LHC) and *V*_oc_. LHC and *V*_oc_ can be determined using eqn (7) and (8), respectively:^40^

7 LHC = 1 − 10^−*f*_osc_^,

8 *V*_oc_ = *E*_LUMO_ − *E*_CB_.

In this context, *f*_osc_ represents the oscillator strength in respect of maximum absorption wavelength, while *E*_LUMO_ and *E*_CB_ denote the energies of the LUMO and conduction band (CB) of the semiconductor.

According to eqn (6), *J*_sc_ can also be increased by enhancing *ϕ*_inject_, which is directly linked to the electron injection driving force (Δ*G*^inj^) from the dye's excited states to the TiO_2_ surface. Generally, a higher Δ*G*^inj^ results in greater *ϕ*_inject_ values. Calculating Δ*G*^inj^ is essential for analyzing photovoltaic data, and it can be determined using eqn (9):^41^

9 Δ*G*^inj^ = ESOP − *E*_CB_.

In eqn (9), ESOP denotes the excited state oxidation potential of the dye, defined as the energy difference between the ground state oxidation potential (GSOP) and the first vertical excitation energy (*E*_g_). For effective electron injection, the dye's ESOP should be above the TiO_2_ conduction band (CB) at −4.05 eV; if it is lower, electron injection may be unfavorable, risking dye elimination. ESOP values can be calculated as:^40^

10 ESOP = GSOP + *E*_g_,

where, the term GSOP represents the energy difference between a neutral species and its oxidized ground state, as defined by the equation in ref. 40:

11 GSOP = *E*^o^(*M*^o^) − *E*^+^(*M*^o^).

For optimal performance, the dye's GSOP must be below the electrolyte's redox potential, *E*^redox^(I^−^/I_3_^−^) (−4.85 eV). The dye regeneration driving force (Δ*G*^reg^) can be calculated using eqn (12):^41^

12 Δ*G*^reg^ = *E*^redox^(I^−^/I_3_^−^) − GSOP.

The charge transfer rate (*k*_CT_) is a key parameter influenced by the π-stacking arrangement of adjacent dyes. The reorganization energy (*λ*) is related to *k*_CT_ as outlined in eqn (13):^40^13
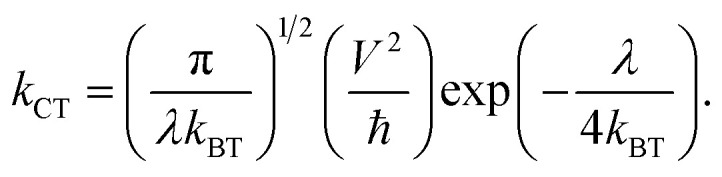
Here, *T*, ℏ, *k*_B_, and *V* represent the absolute temperature, reduced Planck's constant, Boltzmann constant, and electronic coupling matrix element between adjacent dyes, respectively. The *V* values for holes (*V*_+_) and electrons (*V*_−_) are calculated as follows:14
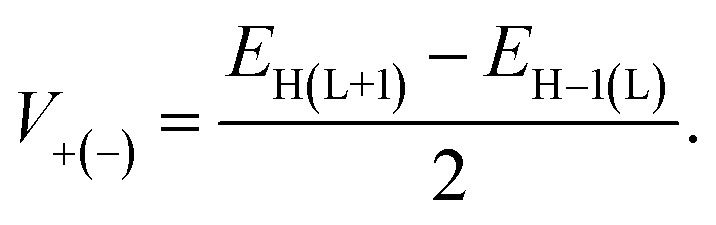
In this context, *E*_H_, *E*_H−1_, *E*_L_, and *E*_L+1_ refer to the energies of the HOMO, HOMO−1, LUMO, and LUMO+1 for the closed-shell neutral dye configuration. The hopping mobility (*μ*_hop_) quantifies the dye molecule's electron or hole transport capabilities and can be calculated using eqn (15):15
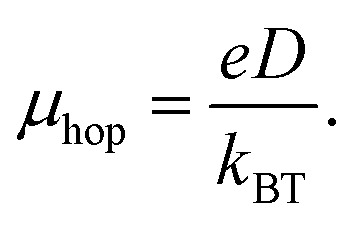
In this context, *e* represents the electronic charge and *D* the diffusion coefficient. The *D* value can be calculated as follows:16
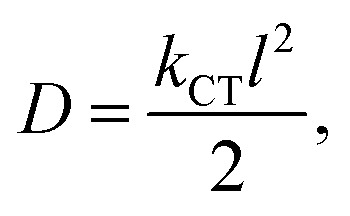
where, *l* refers to the spacing between two adjacent dyes.

## 4 Results and discussion

### 4.1 Dihedral angles

Based on the ground-state optimized geometries, we examined the dihedral angles between the donor and π bridge (D–π), π bridge and acceptor (π–A), and acceptor and anchoring group (A–A′) in all the designed dyes, as these angles critically influence the molecular planarity, conjugation, and charge transfer properties. The detailed values are provided in Table S3 of the SI.

Our analysis indicates that the D–π dihedral angles show the largest variations among the dyes, suggesting that the donor units predominantly govern molecular twisting. In contrast, the π–A and A–A′ angles remain relatively small across the series, implying that the acceptor and anchoring groups exert a comparatively minor influence on structural distortion. This trend is observed consistently across most dyes, although exceptions such as COU–QN exhibit slightly larger deviations.

These geometric variations are not just structural features but also play a key role in determining how efficiently the dyes absorb light and transfer charge. When the D–π linkage is nearly planar, the electron density can delocalize smoothly along the conjugated backbone, which enhances intramolecular charge transfer and shifts absorption toward longer wavelengths. In contrast, twisted linkages interrupt conjugation and weaken light harvesting. The consistently small A–A′ angles further suggest that most dyes are structurally well suited for strong coupling with TiO_2_, promoting faster and more efficient electron injection. Together, these observations highlight that molecular planarity is a vital design factor for improving light absorption and charge transport in D–π–A–A′ dyes.

### 4.2 Frontier molecular orbital (FMO) analysis

To investigate the electron transfer process in the D–A system, it is essential to understand the electronic distribution within the molecular structure. FMO analysis serves as a good tool for predicting the electronic transition behavior and excitation properties of a particular dye.^16^ We have calculated the energies of HOMOs and LUMOs for all the designed dyes, including their respective *Δ*_H–L_ values. These results are detailed in Table 1.

**Table 1:** Energies of HOMOs and LUMOs for designed dyes along with their corresponding *Δ*_H–L_ values

Dyes	HOMO (eV)	LUMO (eV)	*Δ*_H–L_ (eV)
COU–QN	−5.676	−2.840	2.836
TPA–PY	−5.273	−3.384	1.889
IN–PTM	−5.487	−3.080	2.407
CAR–BTZ	−5.552	−3.126	2.426
DPA–NDI	−5.321	−3.635	1.686
THQ–BZ	−5.278	−2.654	2.624
TAT–BZ	−5.172	−2.648	2.524
TPA–PYQ	−4.932	−3.258	1.674
AZ–QN	−5.099	−2.813	2.286
JUD–BTZ	−5.008	−3.029	1.979

While all values are compiled in a single table for ease of comparison, special attention was given to the choice of donor and acceptor fragments during dye design, which directly influences the observed electronic trends.

All the designed dyes meet the essential requirements for efficient operation: they exhibit a narrowed bandgap, a HOMO level below the redox potential of the I^−^–I_3_^−^ electrolyte (−4.8 eV), and a LUMO level above the conduction band of TiO_2_ (−4.0 eV), which together ensure both effective electron injection and dye regeneration. As summarized in Table 1, analysis of the *Δ*_H–L_ values shows that dyes containing strong electron-donating groups (*e.g.*, TPA, DPA, JUD) paired with strongly electron-withdrawing acceptors (*e.g.*, NDI, PYQ) consistently exhibit reduced HOMO–LUMO gaps and favorable orbital alignments. This highlights the key roles of donor and acceptor units, where the donor strength primarily determines the HOMO level and the acceptor strength governs LUMO stabilization.

In summary, the dye library was carefully designed by varying donor and acceptor fragment combinations, and the reported electronic properties reflect these intentional modifications. Dyes such as TPA–PYQ, DPA–NDI, and JUD–BTZ stand out with favorable orbital energies, suggesting improved light-harvesting capabilities and enhanced photocurrent generation in DSSC applications. Overall, these structure–property relationships reinforce a general design principle: balancing donor and acceptor units is an effective strategy for tuning both efficiency and stability.

**Fig. 2:**
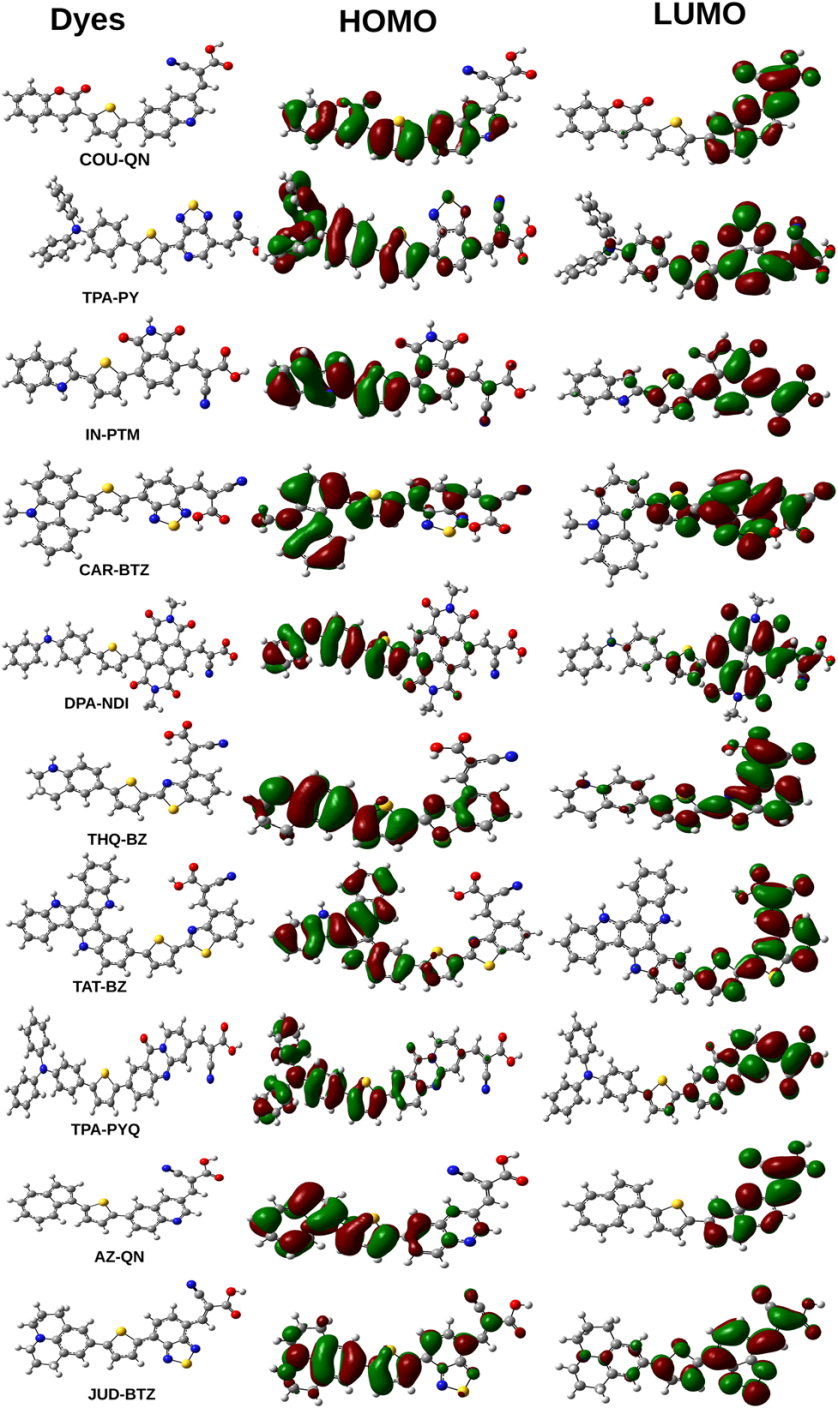
Plot of FMOs for the designed dyes.

We have depicted the distribution of FMOs for the designed dyes in Fig. 2. As shown in this figure, the HOMOs are delocalized across the donor, π-bridging units, and extend into the acceptor unit. Conversely, the LUMOs are delocalized over the entire acceptor unit and anchoring group and extending into the π-bridge unit. Thus, Fig. 2 suggests that all of our designed dyes contribute significant intramolecular charge transfer characteristics.

### 4.3 Reduced density gradient *i.e.*, RDG analysis

**Fig. 3:**
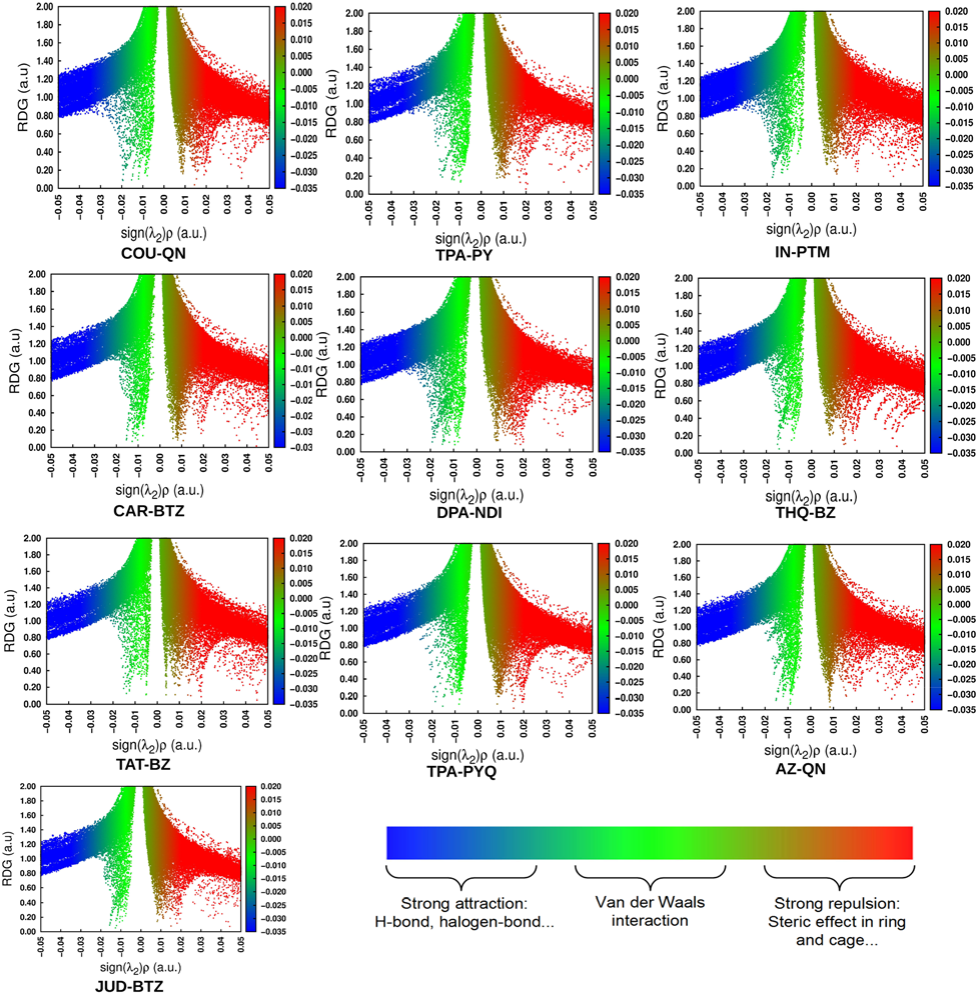
Plot of RDG for the designed dyes.

The RDG graph is a 2D scattered map that shows different interactions within a dye molecule. By analyzing the RDG graph, one can gain insight into the non-covalent interactions. This kind of interactions encompass hydrogen bonding, steric interactions, and van der Waals forces. The RDG data was generated using Multiwfn 3.8 software.^25,42^ Sign(*λ*_2_) is essential for determining the type of interaction present in RDG analysis. An attractive force between the molecules is represented by a negative *λ*_2_. Conversely, a positive *λ*_2_ indicates the existence of repulsive forces. Fig. 3 depicts the 2D scattered graphs for the designed dyes. These graphs display the reduced density gradient on the *y*-axis and the electron density as a function of sign(*λ*_2_) on the *x*-axis. Besides, the colors blue, green, and red represent attractive interactions, van der Waals forces, and strong repulsive interactions, respectively. The negative *λ*_2_ values in RDG graphs are linked to hydrogen bonding and van der Waals forces. These interactions, being more attractive, are known to enhance molecular stability, with strong hydrogen bonding further aiding in the stabilization of molecules. The graphs in Fig. 3 show that attractive and van der Waals forces prevail over repulsive forces, indicating the stability of all the compounds. Furthermore, the graphs indicate that the spikes on both the left and right sides correspond to sign(*λ*_2_) values of −0.05 and +0.05 a.u., respectively. It is observed from Fig. 3 that the blue-colored section on the left side of each plot is positioned higher than the red-colored component on the right side of each plot. This demonstrates that all of our designed molecules are stable, as the attractive forces outweigh the repulsive forces. As a result, the RDG graphs provide a clear indication of the substantial stability attained by the dyes.

### 4.4 Transition density matrix *i.e.*, TDM analysis

Transition Density Matrix (TDM) analysis is a powerful tool for understanding electron localization, electronic excitations, and interactions between donor and acceptor units within a molecule's excited state. In this study, the TDM analysis was performed for the S_0_ → S_1_ transition, corresponding to the first electronic excitation. The TDM was generated from the TD-DFT output using the Multiwfn 3.8 program suite.^42,43^ The numbers shown in the TDM plots represent atomic indices generated in GaussView, which we used to identify individual atoms. Based on these indices, the atoms were grouped into donor (D), π-bridge, internal acceptor (A), and terminal acceptor (A′) segments. Each element of the matrix therefore reflects the extent of transition density overlap between atoms in these different segments, and the “heat” in the TDM plots highlights regions of electron density, revealing the molecule's fragmented structure. The atomic indices generated by GaussView are used solely to assign atoms to structural segments. The analysis focuses on the transition density overlap between these segments rather than individual atoms. A representative diagram showing the atomic indices and their grouping for the COU–QN molecule is provided in the Fig. S2 of SI.

The TDM plots were visualized as heat maps with an isosurface value of 0.002. Positive contributions are shown in green and negative contributions in blue. Hydrogen atoms contribute minimally to the transitions due to the localized nature of their 1s orbitals and are therefore neglected in the analysis.

**Fig. 4:**
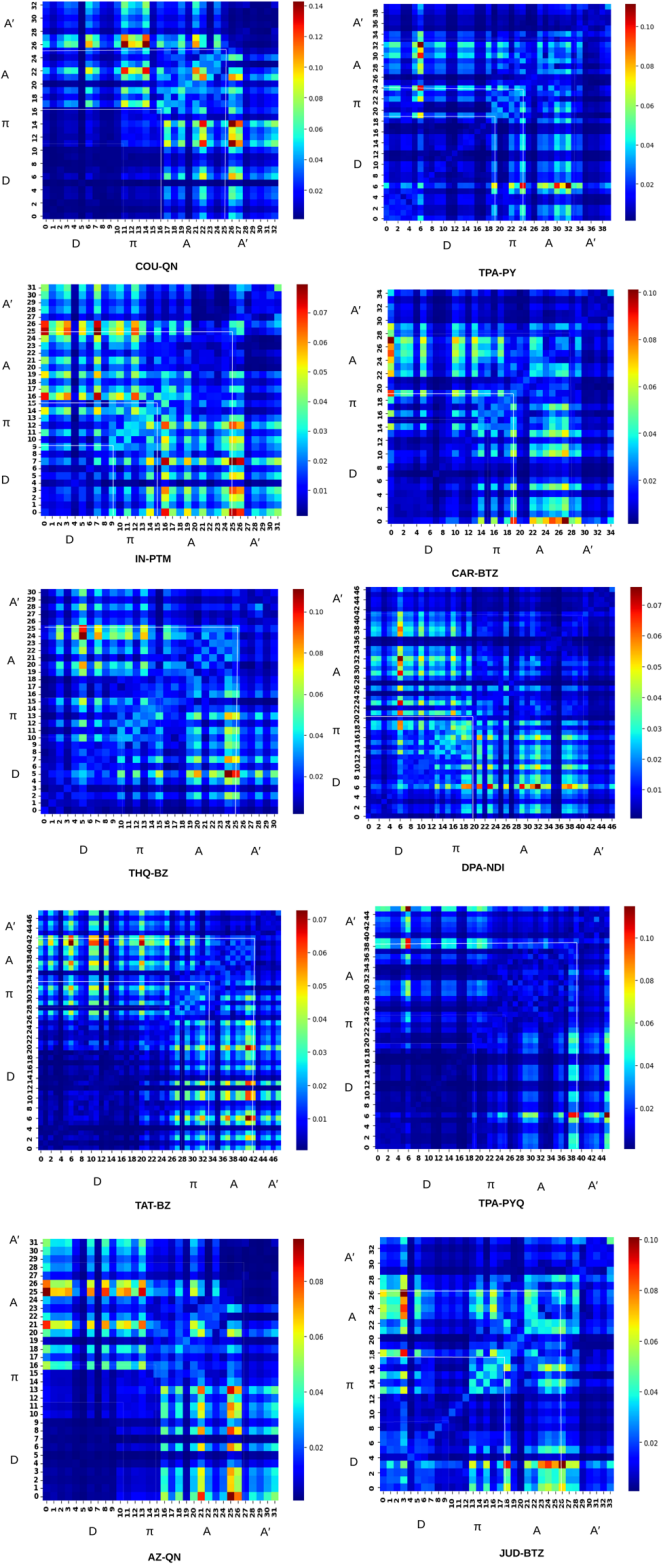
TDM plots for the designed dyes. Here, *x* and *y*-axis correspond to hole and electron position, respectively.

From Fig. 4, it is evident that for each dye, the charge density is effectively distributed, displaying both diagonal and off-diagonal characteristics, with the diagonal capturing the majority of the electron distribution. The analysis also shows that conjugation persists throughout the molecule, facilitating efficient charge transfer from the donor through the π-bridge to the internal and terminal acceptors (A and A′).

### 4.5 Oxidation potential of the dyes

To ensure efficient electron injection and dye regeneration, it is important that the frontier orbitals of the dye and the semiconductor are properly aligned.^44^ In addition, to gain deeper insight into the charge transfer mechanism, we also calculated the free energy changes for dye regeneration (Δ*G*^reg^) and electron injection (Δ*G*^inj^). These parameters were obtained using eqn (9)–(12), and the resulting values are summarized in Table S4 of the SI.

**Fig. 5:**
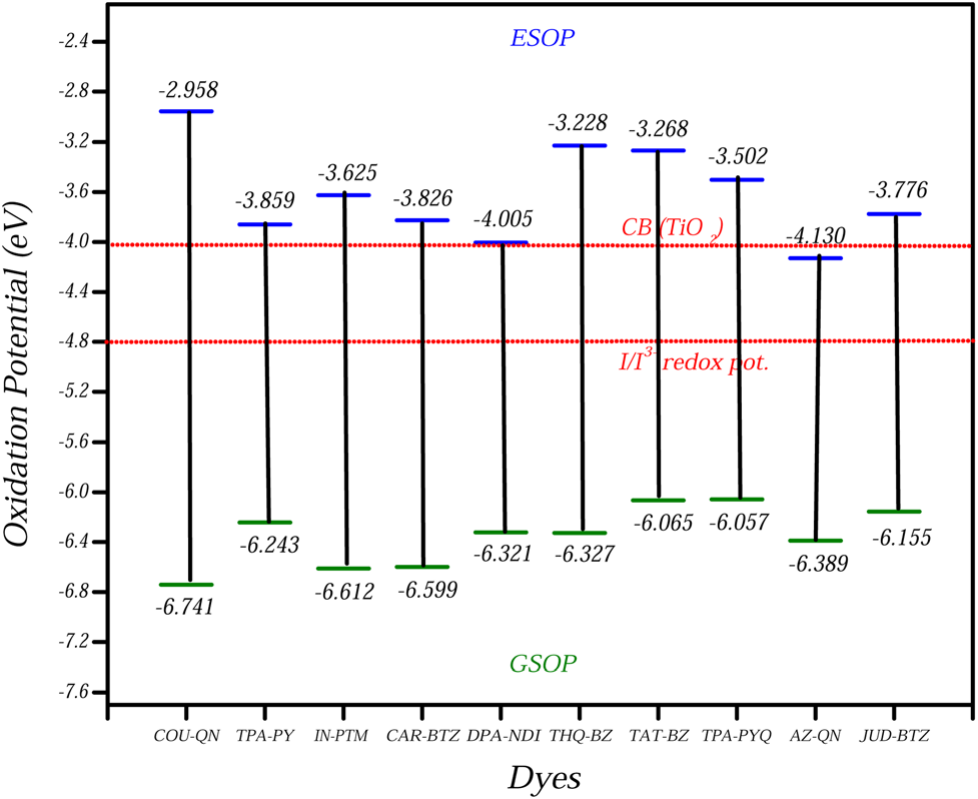
Plot of band-alignment of dyes relative to the CB of TiO_2_ and redox potential of I^−^/I_3_^−^.

The band alignment of the designed dyes relative to the conduction band of TiO_2_ and the redox potential of the I^−^/I_3_^−^ electrolyte is illustrated in Fig. 5. The results show that the GSOP values of all designed dyes lie below the redox potential of the I^−^/I_3_^−^ couple (around −4.85 eV), which indicates that dye regeneration by the electrolyte is energetically favorable. In contrast, nearly all dyes exhibit ESOP values above the conduction band of TiO_2_ (approximately −4.05 = eV), except for AZ–QN. This alignment confirms that the dyes possess sufficient driving forces for both electron injection and dye regeneration. It is evident that the donor strength plays a major role in tuning both GSOP and ESOP values. Dyes containing stronger electron donating groups, such as TPA and DPA, exhibit slightly higher HOMO levels, which translate to lower GSOP values. This facilitates easier oxidation and faster regeneration of the oxidized dye by the redox electrolyte. Conversely, dyes with weaker donors such as COU or CAR possess deeper HOMO levels, resulting in higher GSOP values and a comparatively slower regeneration tendency. Thus, the donor unit directly influences how efficiently a dye can regain electrons after photoexcitation. The nature of the acceptor unit also influences the electron injection process. Strongly electron withdrawing groups such as NDI and PYQ stabilize the LUMO and consequently lower the ESOP, which increases the thermodynamic driving force for electron injection into the TiO_2_ conduction band. In contrast, weaker acceptors such as BZ and QN raise the LUMO level, thereby slightly reducing the injection efficiency. This trend highlights how acceptor design directly governs the interfacial charge transfer behavior of the dyes.

A higher value of Δ*G*^inj^ generally indicates a stronger thermodynamic driving force for electron injection. As seen from Table S4 of the SI, most of the designed dyes exhibit favorable Δ*G*^inj^ values, which supports their suitability for efficient electron transfer to the semiconductor. Similarly, the Δ*G*^reg^ values listed in Table S4 suggest that all dyes are thermodynamically capable of undergoing fast regeneration after photoexcitation. These findings together imply that the designed dyes show strong potential for balanced electron injection and regeneration, both of which are essential for efficient DSSC operation.

The electron injection and dye regeneration processes can also be interpreted in terms of the ionization potential (IP) and electron affinity (EA), which directly influence the energy barriers for charge transfer. Previous studies have shown that for optimal DSSC performance, strong charge injection and efficient charge transport must be accompanied by a proper balance between hole and electron mobilities.^40,45^ A lower IP facilitates the removal of electrons and promotes hole generation, while a lower EA favors electron transfer from the dye to the semiconductor. The calculated IP and EA values, obtained using eqn (1) and (2), are listed in Table S5 in SI. These results further support that the designed dyes possess energetically favorable conditions for efficient photoinduced charge separation and transport. The data in Table S5 of SI reveals that the TPA–PYQ dye exhibits the lowest calculated IP value, suggesting a strong preference for hole formation and dye regeneration processes. In contrast, the TAT–BZ dye shows the lowest EA value, indicating its minimal tendency to accept electrons. Consequently, the transfer of electrons to the conduction band (CB) of the TiO_2_ semiconductor surface is facilitated most efficiently by the TAT–BZ dye.

### 4.6 Absorption properties of the dyes

To attain insights into the absorption properties of the designed dyes, we have depicted the excitation properties for 30 excited states, with the dominant electronic transitions reported in Table 2. Furthermore, Table 2 presents the values of parameters including *E*_g_, *λ*_max_, *f*_osc_, electronic transitions, LHC, *μ*, and *V*_oc_.

**Table 2:** *E*_g_, *λ*_max_, *f*_osc_, electronic transitions, LHC, *μ* and *V*_oc_ for the designed dyes

Dyes	*E*_g_ (eV)	*λ*_max_ (nm)	*f*_osc_	Transitions	LHC	*μ* (debye)	*V*_oc_
COU–QN	3.78	328	0.93	H → L (86.49%)	0.88	5.11	2.38
TPA–PY	2.38	520	1.24	H → L (77.01%)	0.94	10.53	1.51
IN–PTM	2.99	415	1.19	H → L (72.71%)	0.93	3.00	1.96
CAR–BTZ	2.77	447	0.86	H → L (63.79%)	0.86	10.91	1.85
DPA–NDI	2.32	535	0.61	H → L (77.50%)	0.75	9.14	1.28
THQ–BZ	3.10	400	1.06	H → L (69.27%)	0.91	12.11	2.38
TAT–BZ	2.80	443	0.16	H → L (78.38%)	0.30	7.34	1.93
TPA–PYQ	2.55	485	0.77	H → L (44.35%)	0.83	1.89	1.77
AZ–QN	2.26	549	0.01	H → L (88.04%)	0.02	4.42	2.30
JUD–BTZ	2.38	521	1.08	H → L (84.58%)	0.92	8.57	1.86

**Fig. 6:**
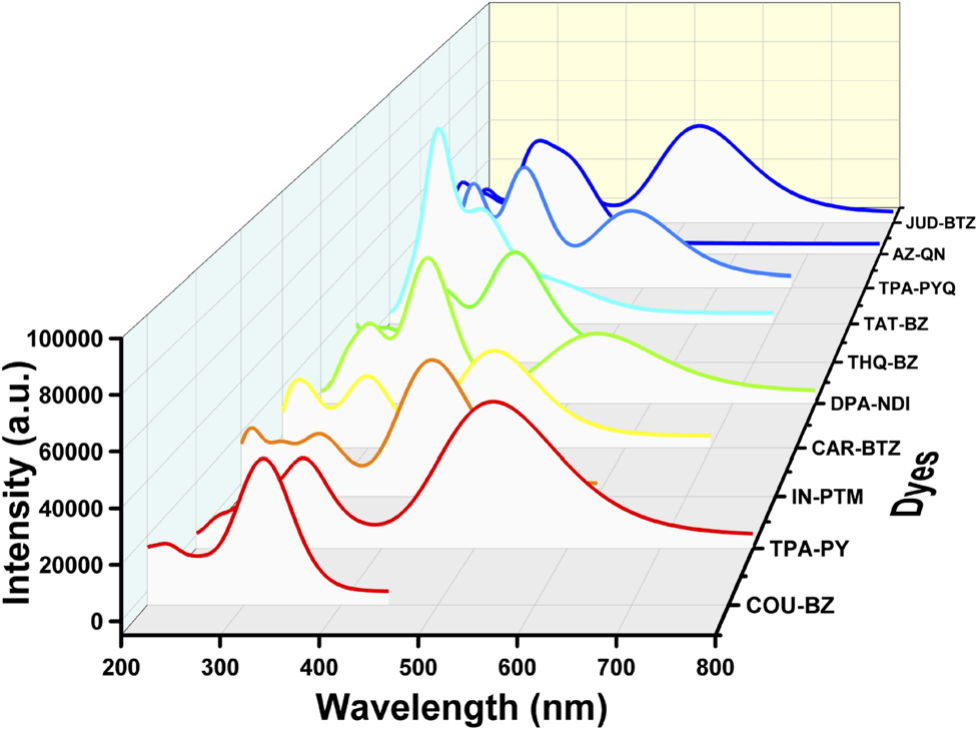
UV-visible spectra of the designed dyes.

Analysis of Table 2 reveals that dyes TPA–PY, DPA–NDI, AZ–QN, and JUD–BTZ exhibit higher *λ*_max_ values, indicating strong visible-light absorption. Dyes such as TPA–PY, IN–PTM, THQ–BZ, and JUD–BTZ also show relatively higher oscillator strengths (*f*_osc_), suggesting enhanced light-harvesting capacities (LHC). The corresponding spectra are provided in Fig. 6.

Eqn (6) indicates that higher LHC values correlate with greater short-circuit current densities (*J*_sc_), which contribute to improved power conversion efficiency. Table 2 further shows that all designed dyes exhibit comparatively higher *V*_oc_ values, implying favorable energy alignment with the TiO_2_ conduction band. The observed absorption characteristics are primarily governed by molecular structure: dyes with extended π-conjugation (*e.g.*, TPA–PY, DPA–NDI, and JUD–BTZ) exhibit red-shifted absorption, while rigid fused-ring systems such as COU–QN and THQ–BZ show blue-shifted absorption. Strong donor moieties (TPA, DPA) enhance oscillator strength and light absorption, whereas strong acceptors (NDI, QN) stabilize the excited states and promote red-shifted spectra. Additionally, larger transition dipole moments (*μ*) observed in dyes such as THQ–BZ, CAR–BTZ, and TPA–PY suggest efficient charge separation and electron injection into TiO_2_.

To gain deeper insights into how charge is transported in our designed dyes, we have calculated the reorganization energies (*λ*) and reported them in Table S6 of SI. For efficient charge transport, it is crucial to achieve lower values of *λ* (*λ*_+_ or *λ*_−_). A lower *λ*_−_ indicates enhanced electron transport capability in the designed dye. Conversely, a lower *λ*_+_ signifies the hole transport characteristics of the designed dyes. From Table 6, it is evident that for the designed dyes IN–PTM, CAR–BTZ, TAT–BZ, TPA–PYQ, and AZ–QN, the *λ*_+_ values are lower than the *λ*_−_ values. This suggests efficient hole transportation in these dyes. In contrast, for COU–QN, TPA–PY, DPA–NDI, THQ–BZ, and JUD–BTZ, the *λ*_−_ values are smaller than the *λ*_+_ values, indicating enhanced electron transportation in these dyes.

We have also calculated the total reorganization energy (*λ*_tot_) values and they are also listed in Table S6. *λ*_tot_ represents the sum of *λ*_+_ and *λ*_−_. For effective electron–hole separation, the *λ*_tot_ values of the dyes should be low in order to reduce recombination processes.^40^ From Table 6, it is noted that COU–QN, THQ–BZ, TAT–BZ, and JUD–BTZ exhibit relatively lower *λ*_tot_ values compared to the other designed dyes. This indicates better efficiency in electron–hole separation and suggests potentially slower recombination processes in these dyes.

To determine the electronic coupling matrix element (*V*), we have analyzed the π-stacking arrangement of two adjacent dyes. The calculated values of *V* (using eqn (14)) are presented in Table 3. With these *V* values, we have derived the *k*_CT_ values for holes (*k*_CT_^+^) and electrons (*k*_CT_^−^) (using eqn (13)), which are also presented in Table 3.

**Table 3:** *V*, *k*_CT_, and *μ*_hop_ values of the designed dyes for both holes and electrons

Dyes	*V*_+_ (eV)	*V*_−_ (eV)	*k*_CT_^+^ × 10^14^ (s^−1^)	*k*_CT_^−^ × 10^14^ (s^−1^)	*l* (Å)	*μ*_hop_^+^ (cm^2^ V^−1^ s^−1^)	*μ*_hop_^−^ (cm^2^ V^−1^ s^−1^)
COU–QN	0.062	0.094	0.482	2.003	3.5	1.142	4.745
TPA–PY	0.209	0.019	0.023	0.029	3.5	0.054	0.069
IN–PTM	0.099	0.114	1.278	0.865	3.5	3.028	2.049
CAR–BTZ	0.133	0.118	4.333	0.054	3.5	10.265	0.128
DPA–NDI	0.855	0.179	4.999	2.034	3.5	11.843	4.819
THQ–BZ	0.432	0.259	23.996	8.750	3.5	56.850	20.730
TAT–BZ	0.138	0.407	6.124	17.458	3.5	14.509	41.361
TPA–PYQ	0.036	0.267	0.181	1.707	3.5	0.429	4.044
AZ–QN	0.057	0.333	0.261	0.054	3.5	0.618	0.128
JUD–BTZ	0.213	0.191	4.255	4.506	3.5	10.081	10.675

As summarized in Table 3, the dyes naturally divide into two groups based on their charge-transfer rate constants. IN–PTM, CAR–BTZ, DPA–NDI, THQ–BZ, and AZ–QN exhibit higher *k*_CT_^+^ values, suggesting a preference for hole transport, while the others show larger *k*_CT_^−^ values, favoring electron transport. This division is also reflected in the hopping mobilities: the former set displays relatively higher *μ*_hop_^+^, whereas the latter group demonstrates larger *μ*_hop_^−^, pointing to more efficient electron migration.

These patterns align closely with the structural makeup of the dyes. Donor-rich frameworks, particularly those containing TPA and DPA units, stabilize hole transport by raising the HOMO energy and lowering the reorganization energy (*λ*_+_), which promotes smooth charge regeneration. Acceptor-rich dyes such as QN- and NDI-based systems, in contrast, stabilize electron transport by lowering *λ*_−_, which enhances electron mobility. Extended π-conjugation in molecules like DPA–NDI and THQ–BZ further strengthens intermolecular π–π interactions, increasing the electronic coupling (*V*) and consequently boosting the hopping mobility. On the other hand, rigid fused-ring designs such as COU–QN and TAT–BZ limit charge delocalization, which restrains mobility but keeps reorganization energies relatively low.

Overall, these results demonstrate that the charge-transport properties of the designed dyes are not random but follow clear structure–property relationships. By tuning the donor, acceptor, and π-bridge components, one can intentionally bias a dye toward hole or electron transport, or design for balanced behavior. Such insights are valuable for tailoring dye frameworks to achieve efficient charge separation and controlled carrier transport in DSSCs.

### 4.7 Adsorption on TiO_2_ surface

#### 4.7.1 Analysis of FMO

**Fig. 7:**
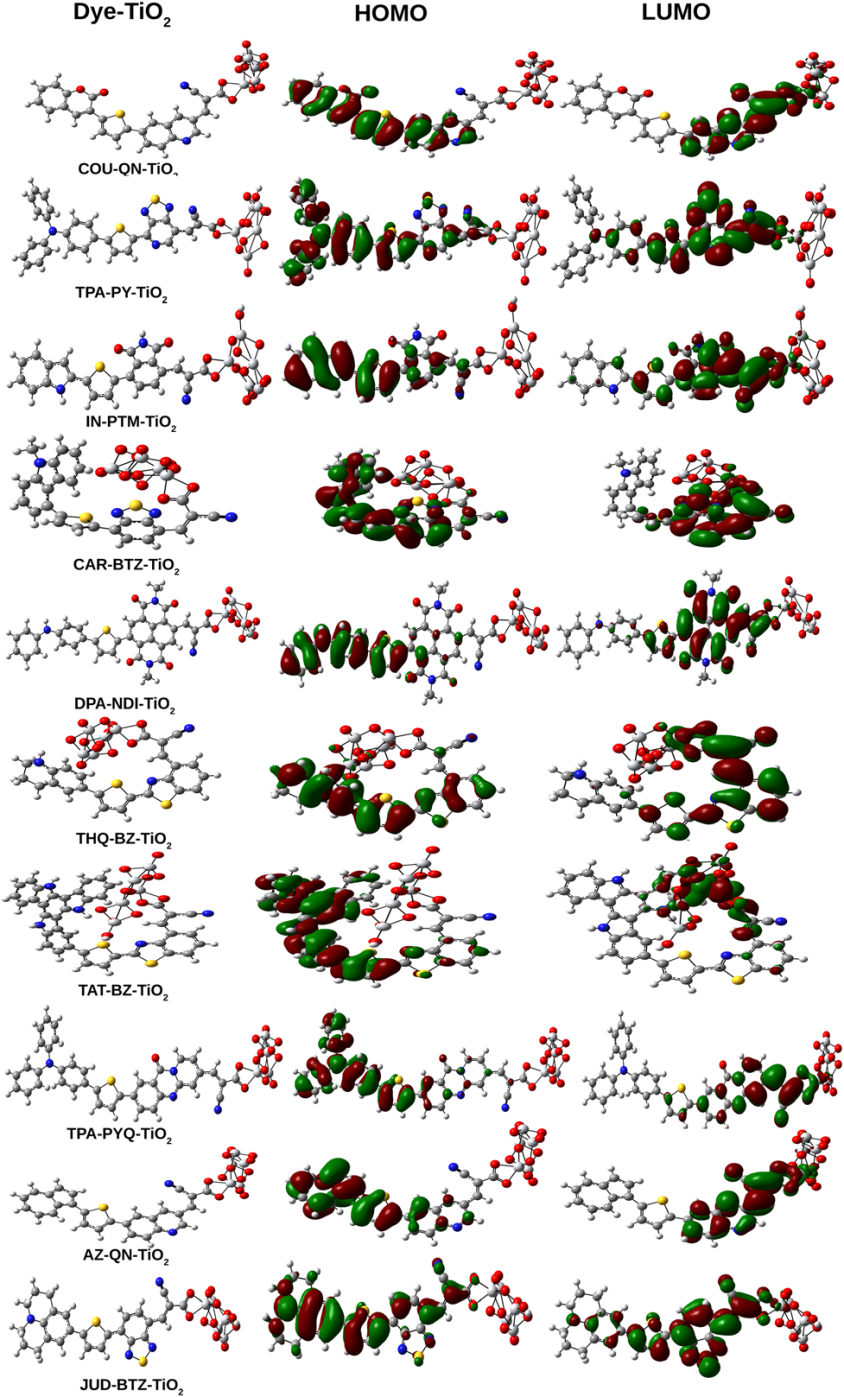
Optimized geometries of the dye–Ti_5_O_10_ clusters along with their FMOs.

To accurately model the performance of a more realistic solar cell device, it is crucial to analyze the optical properties of the designed dyes. For this purpose, we have employed a Ti_5_O_10_ cluster to emulate the TiO_2_ semiconductor surface. The selection of Ti_5_O_10_ cluster to mimic the TiO_2_ semiconductor surface has been justified in the Table S3 of SI. Cyanoacrylic acid was utilized as the anchoring group to facilitate dye adsorption onto the semiconductor surface. Fig. 7 displays the optimized structures of the dye–Ti_5_O_10_ clusters alongside their Frontier Molecular Orbitals (FMOs). In this figure, the HOMOs exhibit delocalization across the donor unit, the π-bridging unit, and the acceptor unit, with additional contribution from the anchoring group. Conversely, the LUMOs are delocalized over the acceptor unit and anchoring group, with some contribution from the π-bridging unit and the Ti_5_O_10_ cluster, as illustrated.

We have also calculated the *Δ*_H–L_ and *μ* values for the designed dye–Ti_5_O_10_ clusters, which are summarized in Table 4. Analysis of this table reveals that the *Δ*_H–L_ values for the isolated dyes are greater than those for the dye–Ti_5_O_10_ clusters. Furthermore, upon comparing Table 4 with Table 2, it is evident that the *μ*_g_ values of the dye–Ti_5_O_10_ clusters exceed those of the isolated dyes. This proves the enhancement of the charge transport properties of the designed dyes upon binding it to the Ti_5_O_10_ semiconductor surface.

**Table 4:** *Δ*_H–L_, *μ*_g_, *E*_1_ and *E*_b_ values for the dye–Ti_5_O_10_ clusters

Dye–Ti_5_O_10_	HOMO (eV)	LUMO (eV)	*Δ*_H–L_ (eV)	*μ*_g_ (debye)	*E*_1_ (eV)	*E*_b_ (eV)
COU–QN–Ti_5_O_10_	−5.839	−3.444	2.395	13.277	2.028	0.367
TPA–PY–Ti_5_O_10_	−5.421	−3.638	1.783	17.775	1.659	0.124
IN–PTM–Ti_5_O_10_	−5.693	−3.612	2.081	11.950	1.871	0.210
CAR–BTZ–Ti_5_O_10_	−6.387	−3.094	3.293	15.897	2.804	0.489
DPA–NDI–Ti_5_O_10_	−5.373	−3.807	1.566	13.566	1.366	0.200
THQ–BZ–Ti_5_O_10_	−5.965	−3.094	2.871	14.881	2.466	0.405
TAT–BZ–Ti_5_O_10_	−5.414	−3.557	1.857	15.369	1.460	0.397
TPA–PYQ–Ti_5_O_10_	−5.055	−3.774	1.281	12.564	1.148	0.133
AZ–QN–Ti_5_O_10_	−5.240	−3.432	1.808	12.644	1.496	0.312
JUD–BTZ–Ti_5_O_10_	−5.291	−3.498	1.793	20.764	1.726	0.067

#### 4.7.2 Analysis of RDG graph for dye–clusters

**Fig. 8:**
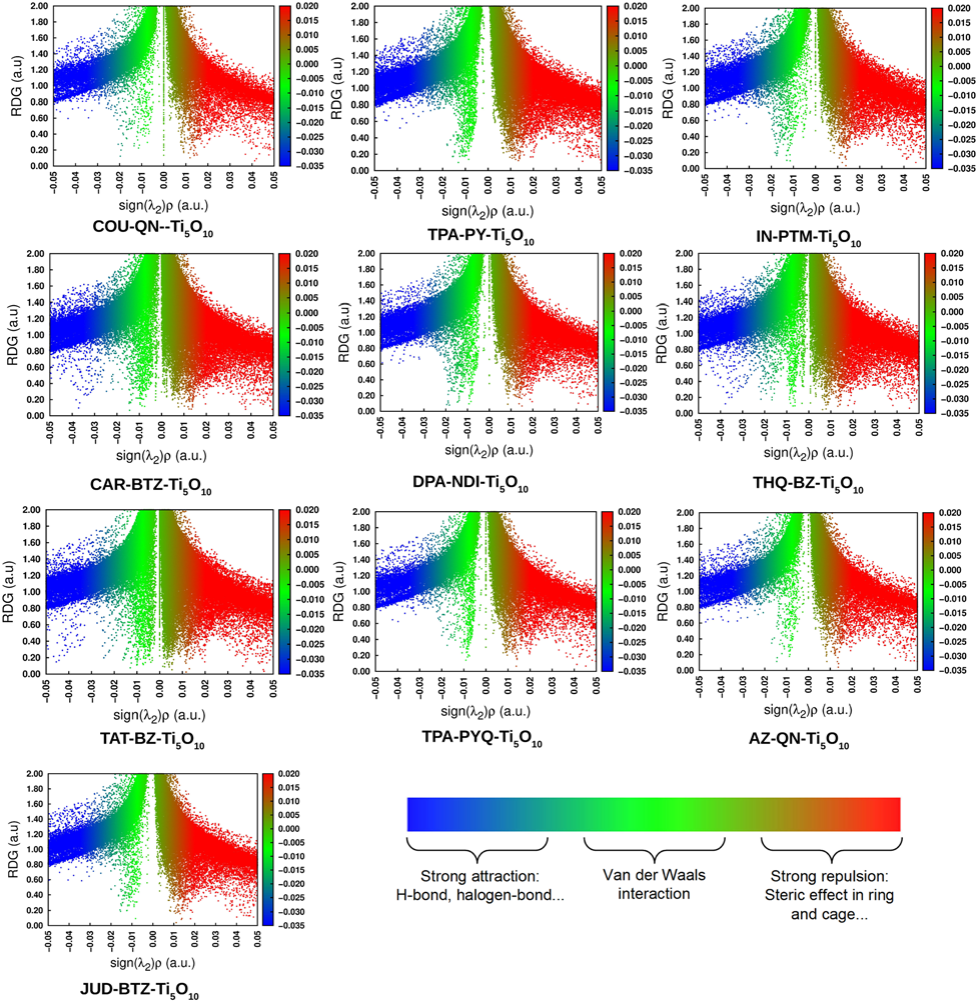
Plot of RDG for the dye–clusters.

The RDG graph *i.e.*, a 2D scattered map provides insight into the non-covalent interactions present in a molecule. This kind of interactions include steric interactions, van der Waals forces, and hydrogen bonding. The Multiwfn 3.8 program was used to generate all of the data for the RDG study. The plot of the 2D scattered graphs for the dye–clusters is shown in Fig. 8. The spikes on both the left and right sides of the graphs correspond to sign(*λ*_2_) values of −0.05 and +0.05 a.u., respectively. It is clear from Fig. 8 that each plot's blue-colored portion on the left side is positioned higher than its red-colored component on the right. This shows that the attractive forces dominate the repulsive forces in all of our dye–clusters, indicating their stability. Thus, the RDG graphs give a clear picture of the significant stability that the dye–clusters were able to achieve. As a result, the RDG graphs clearly illustrate the notable stability that the dye–clusters were able to attain.

#### 4.7.3 Dye–clusters' Ti–O bond length and adsorbed dyes' adsorption energy on TiO_2_ semiconductor surface

**Fig. 9:**
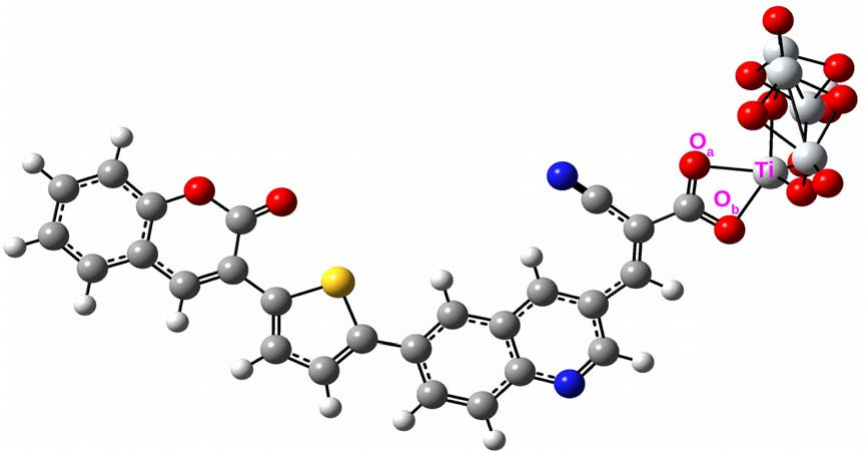
Representation of the Ti–O bond lengths in COU–QN–Ti_5_O_10_ cluster.

Dye adsorption is often indicated by a change in the bond length between the Ti atom of the TiO_2_ semiconductor surface and the O atom of dye. In this regard, we have determined the Ti–O bond lengths for the designed dye–Ti_5_O_10_ clusters, which are listed in Table 5. Fig. 9 illustrates a representation of the Ti–O bond lengths specifically for the COU–QN–Ti_5_O_10_ cluster.

**Table 5:** Ti–O bond lengths, energy of dye–TiO_2_ (*E*_dye+TiO_2__), energy of dye (*E*_dye_), energy of TiO_2_ (*E*_TiO_2__), and adsorption energy (*E*_ads_) for the dye–clusters

Dye–Ti_5_O_10_	Ti–O_a_ (Å)	Ti–O_b_ (Å)	*E*_dye+TiO_2__ (eV)	*E*_dye_ (eV)	*E*_TiO_2__ (eV)	*E*_ads_ (eV)
COU–QN–Ti_5_O_10_	2.036	2.032	−77595.177	−49194.350	−28396.267	−4.560
TPA–PY–Ti_5_O_10_	2.034	2.038	−94072.020	−65674.251	−28396.267	−4.502
IN–PTM–Ti_5_O_10_	2.037	2.032	−76995.305	−48594.437	−28396.267	−4.601
CAR–BTZ–Ti_5_O_10_	2.035	2.072	−88386.749	−59984.796	−28396.267	−5.686
DPA–NDI–Ti_5_O_10_	2.032	2.039	−95171.899	−66771.254	−28396.267	−4.378
THQ–BZ–Ti_5_O_10_	2.030	2.088	−83803.102	−55400.969	−28396.267	−5.866
TAT–BZ–Ti_5_O_10_	2.061	2.033	−102403.434	−74003.126	−28396.267	−4.041
TPA–PYQ–Ti_5_O_10_	2.038	2.031	−91135.998	−62735.504	−28396.267	−4.227
AZ–QN–Ti_5_O_10_	2.035	2.031	−74569.898	−46169.038	−28396.267	−4.593
JUD–BTZ–Ti_5_O_10_	2.031	2.031	−87414.857	−59013.995	−28396.267	−4.595

Based on the data presented in Table 5, it is evident that the Ti–O bond lengths for all designed dye–clusters fall within the range of 2.030–2.088 Å. These values are consistent with the Ti–O bond lengths (2.03–2.24 Å) previously reported in theoretical studies.^40,46^ Therefore, this consistency strongly suggests that all designed dyes undergo chemisorption onto the semiconductor surface of Ti_5_O_10_. Looking beyond the numbers, the variation in Ti–O distances and adsorption energies gives a clearer picture of how the dyes actually interact with TiO_2_. For instance, the shorter bonds and stronger adsorption seen in THQ–BZ and CAR–BTZ suggest a tighter anchoring that should help both electron injection and long-term stability. On the other hand, dyes such as TAT–BZ and TPA–PYQ, which bind a bit more weakly, may still perform well in terms of charge transfer but could be more prone to desorption over time. This balance shows that adsorption is not just a structural detail, it plays a direct role in both charge-transfer efficiency and device durability, making anchoring design a key factor for practical DSSCs.

Additionally, in the context of DSSCs, adsorption energy (*E*_ads_) refers to the energy associated with the interaction between the dye molecules and the TiO_2_ semiconductor surface. It is the energy change when dye molecules get adsorbed onto the surface of the semiconductor material. A negative *E*_ads_ indicates that the adsorption process is exothermic *i.e.*, energy is released when the dye molecules bind to the semiconductor surface. This suggests a stable interaction between the dye and the semiconductor, which is generally desirable for efficient DSSCs. *E*_ads_ can be calculated using the formula: *E*_ads_ = *E*_dye+TiO_2__ − (*E*_dye_ + *E*_TiO_2__), where *E*_dye+TiO_2__, *E*_dye_ and *E*_TiO_2__ denote the energies of the dye–Ti_5_O_10_ complex, the isolated dye and the pure Ti_5_O_10_ cluster, respectively.^43,47^ The calculated *E*_ads_ values for each dye–cluster are presented in Table 5. The table shows that all dye–clusters exhibit negative *E*_ads_ values, signifying that the dyes undergo chemisorption onto the TiO_2_ semiconductor surface. This indicates effective electron transfer from the dye's LUMO to the conduction band of TiO_2_.

### 4.8 TDM and exciton binding energy analysis for dye–clusters

**Fig. 10:**
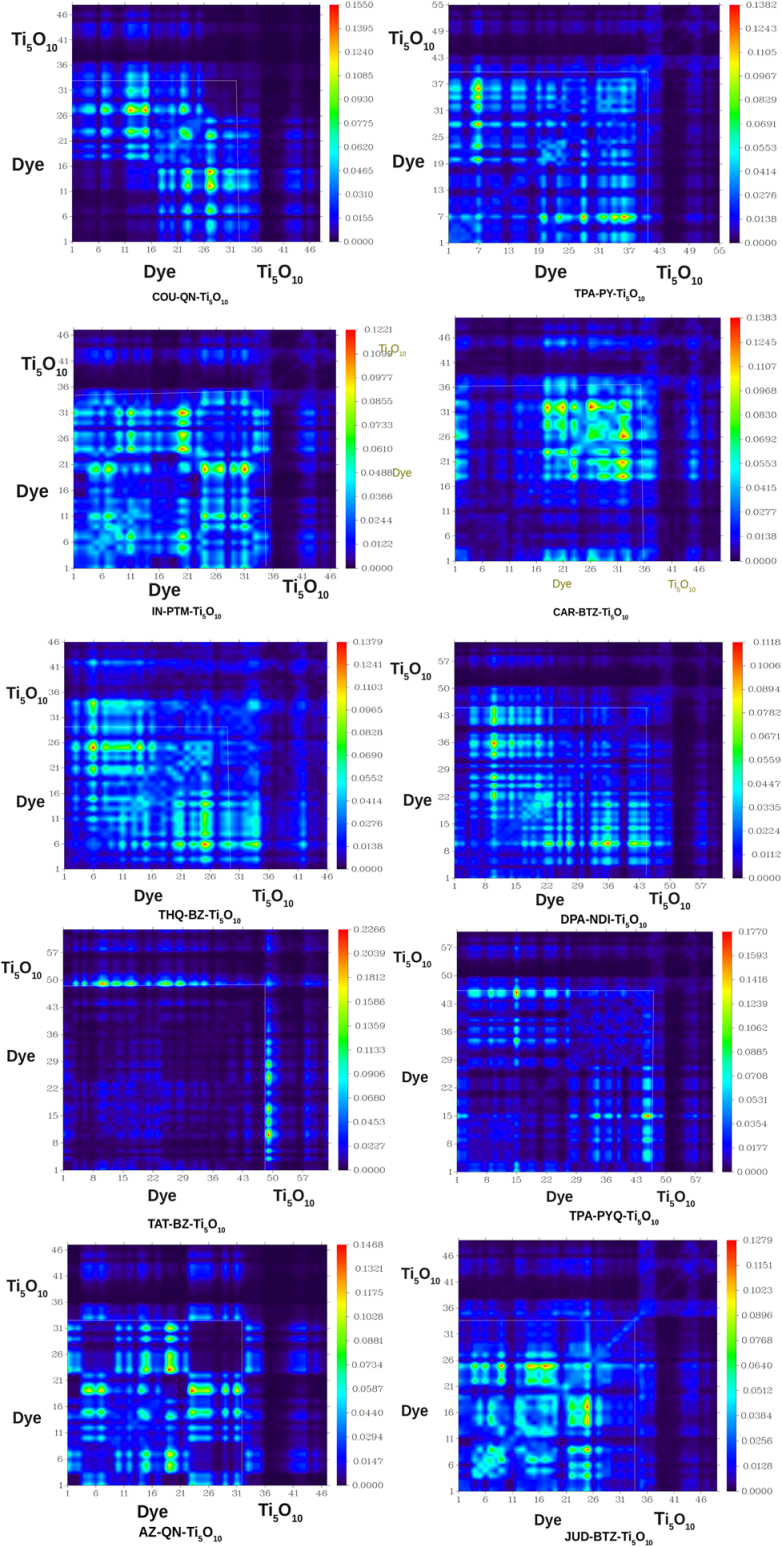
TDM plots for the dye–clusters. Here, *x* and *y*-axis correspond to hole and electron position, respectively.

To analyze the TDM plots, all of the dye–clusters are divided into two groups, *viz.*, dye and Ti_5_O_10_ cluster. The Multiwfn 3.8 program suite is employed to produce TDM plots as heat maps for the dye–clusters in gas phase. Fig. 10 depicts the TDM plots for the dye–clusters with an isosurface value of 0.002. Fig. 10 clearly shows that the charge density is extensively distributed across the dye and the Ti_5_O_10_ cluster, depicted in a diagonal format. The behavior of all the designed dyes has been verified to exhibit efficient electron transfer from the dye to the Ti_5_O_10_ cluster. This visual representation reveals a green area in the dye and a blue area in the Ti_5_O_10_ cluster, highlighting substantial electron transfer from the dye to the TiO_2_ semiconductor surface.

Furthermore, exciton binding energy *i.e.*, *E*_b_ is essential for assessing several properties, such as electronic and optical properties, excited state separation potential, and the performance of DSSCs. The *E*_b_ is the energy needed to separate an electron and a hole within an exciton. A lower *E*_b_ means it's easier for charges to detach from the dye molecule and move to the semiconductor surface. *E*_b_ can be calculated using the formula: *E*_b_ = *Δ*_H–L_ − *E*_1_, where *E*_1_ represents the energy needed for the S_0_ → S_1_ transition.^43,48^ The calculated *E*_b_ values for the dye–clusters are reported in Table 4. This table shows that the *E*_b_ values for the dye–clusters follow this increasing order: JUD–BTZ–Ti_5_O_10_ < TPA–PY–Ti_5_O_10_ < TPA–PYQ–Ti_5_O_10_ < DPA–NDI–Ti_5_O_10_ < IN–PTM–Ti_5_O_10_ < AZ–QN–Ti_5_O_10_ < COU–QN–Ti_5_O_10_ < TAT–BZ–Ti_5_O_10_ < THQ–BZ–Ti_5_O_10_ < CAR–BTZ–Ti_5_O_10_. Thus, JUD–BTZ–Ti_5_O_10_, TPA–PY–Ti_5_O_10_ and TPA–PYQ–Ti_5_O_10_ exhibit a greater capacity for exciton dissociation and efficient charge transfer among all dye–clusters. Additionally, the lower *E*_b_ values of JUD–BTZ–Ti_5_O_10_, TPA–PY–Ti_5_O_10_ and TPA–PYQ–Ti_5_O_10_ contribute to their increased *J*_sc_ values among all the dye–clusters.

#### 4.8.1 Analysis of molecular electrostatic potential surface (MEPS) for dye–clusters

**Fig. 11:**
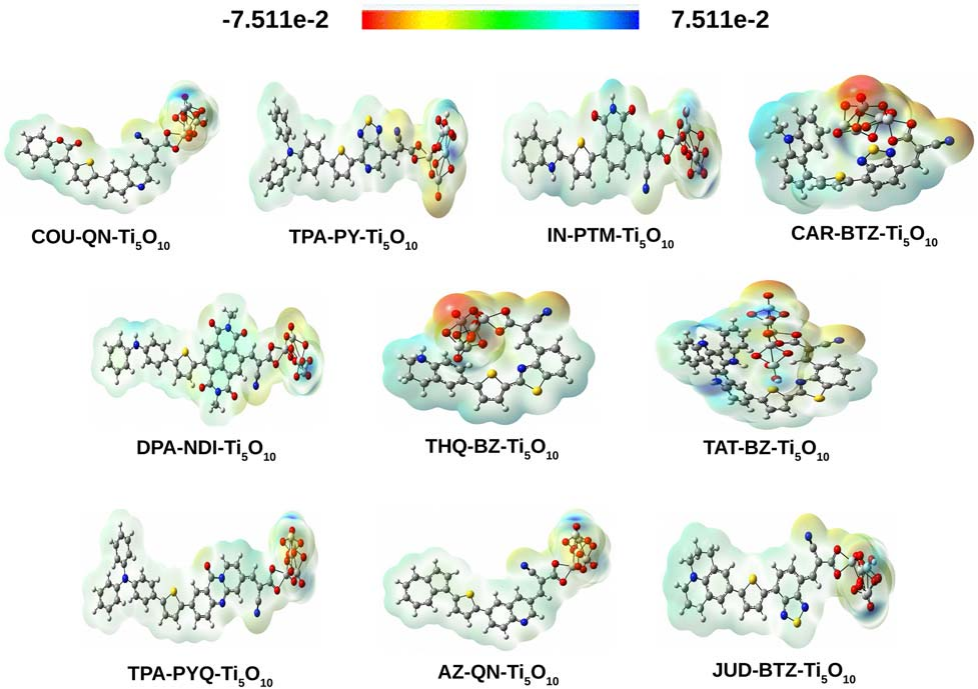
MEPS contour plots for all dye–Ti_5_O_10_ clusters.

The MEPS map visually represents the 3D distribution of molecular charges. It shows where electrons are rich or deficient within the designed dye–clusters, aiding in understanding their interactions. These maps highlight both positive and negative electrostatic potentials within the dye–cluster. Each map uses a color scale to differentiate between negative and positive potentials. In Fig. 11, red indicates regions with the most negative potential, suggesting areas with high electron density. In contrast, blue represents regions with the most positive potential, indicating areas with lower electron density. Interestingly, it has been noted that the designed dye–clusters exhibit identical patterns in the distribution of molecular electrostatic potential. The dyes exhibit a widespread positive electrostatic potential (blue color), while the Ti_5_O_10_ surface shows a widespread negative electrostatic potential (red color). This indicates the substantial charge transfer characteristics present in all studied dye–clusters.

#### 4.8.2 Absorption properties of all dye–Ti_5_O_10_ clusters

**Fig. 12:**
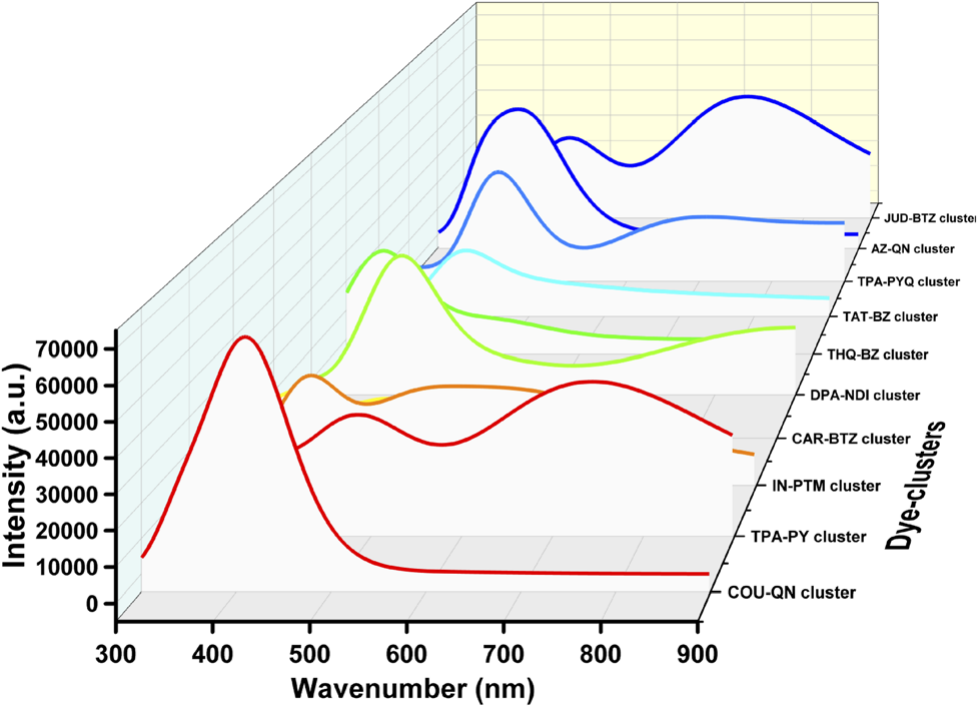
Plot of the UV-visible spectra of all designed dye–Ti_5_O_10_ clusters.

In order to investigate the absorption properties of the dye–Ti_5_O_10_ clusters, we have utilized the CAM-B3LYP functional to estimate the maximum absorption wavelength (*λ*_max_), the excitation energies (*E*_g_), oscillator strengths (*f*_osc_), transitions (FMOs' contribution), dipole moments (*μ*) and LHC. The results are presented in Table 6. This table shows that the studied dye–Ti_5_O_10_ clusters show a decrease in the *E*_g_ and an increase in the *λ*_max_ values. This suggests that the adsorbed dyes undergo red-shift than the isolated dyes. We have presented the plot of the UV-visible spectra of all the designed dyes in Fig. 12.

**Table 6:** *λ*_max_, *f*_osc_, *E*_g_, transitions and *μ* of the dye–Ti_5_O_10_ clusters

Dye–Ti_5_O_10_	*E*_g_ (eV)	*λ*_max_ (nm)	*f*_osc_	Transitions	*μ* (debye)	LHC
COU–QN–Ti_5_O_10_	3.12	397	0.08	H → L (78.77%)	12.90	0.17
TPA–PY–Ti_5_O_10_	2.23	556	1.62	H → L (79.67%)	15.65	0.98
IN–PTM–Ti_5_O_10_	2.70	459	1.34	H → L (80.47%)	10.63	0.95
CAR–BTZ–Ti_5_O_10_	3.42	362	0.28	H−1 → L (66.44%)	15.71	0.47
DPA–NDI–Ti_5_O_10_	2.23	556	0.67	H → L (77.97%)	11.85	0.79
THQ–BZ–Ti_5_O_10_	3.17	391	0.31	H → L (58.48%)	14.83	0.51
TAT–BZ–Ti_5_O_10_	2.55	486	0.05	H−1 → L (65.02%)	10.21	0.11
TPA–PYQ–Ti_5_O_10_	2.23	555	0.86	H → L (50.98%)	10.73	0.86
AZ–QN–Ti_5_O_10_	2.27	547	0.01	H → L+6 (88.86%)	12.14	0.02
JUD–BTZ–Ti_5_O_10_	2.16	574	1.45	H → L (85.96%)	18.45	0.96

Upon analyzing Table 6, it becomes evident that most dye–Ti_5_O_10_ clusters exhibit an increase in their *λ*_max_ compared to the corresponding isolated dyes. However, four exceptions are observed: CAR–BTZ–Ti_5_O_10_, THQ–BZ–Ti_5_O_10_, AZ–QN–Ti_5_O_10_, and JUD–BTZ–Ti_5_O_10_. For the remaining clusters, attachment to the TiO_2_ semiconductor surface induces a shift of *λ*_max_ toward longer, more visible wavelengths. The reduction in the LUMO energies upon cluster formation can be attributed to interactions between the dye's electron-acceptor group (–COOH) and the Ti 3d orbitals.^43^ These interactions account for the observed red-shift in the absorption spectra of the clusters.

A closer look at the results shows that the red-shifts mainly arise from stronger electronic interactions between the dyes and the TiO_2_ cluster. Dyes such as TPA–PY, IN–PTM, and JUD–BTZ not only absorb at longer wavelengths but also exhibit higher oscillator strengths and larger dipole moments, which together promote more effective light harvesting and charge separation at the interface. In contrast, AZ–QN–Ti_5_O_10_, despite having a narrowed gap, shows almost negligible oscillator strength, indicating that not all red-shifted dyes are equally useful for photocurrent generation. This comparison highlights that absorption performance is not dictated by *λ*_max_ alone, but also by how strongly the dye interacts with light and how efficiently it can separate charges. In practice, dyes with a balanced combination of narrow excitation energies, strong absorption, and sizable dipole moments stand out as the most promising candidates for efficient DSSCs.

This study has presented the fundamental electronic and optical features of the designed dyes and proposed several new candidates with favorable alignment for use in DSSCs. These results provide a basic framework that others can build on, using our insights as a starting point for exploring further structural modifications or even new dye architectures. Looking forward, two key application-driven directions stand out: (i) designing dyes that extend absorption deeper into the near-infrared region, and (ii) improving molecular stability under long-term illumination, both of which are essential for higher efficiency and durability. On the methodological side, future computational studies could include solvent polarity, electrolyte composition, and co-adsorbents to more realistically capture interfacial charge-transfer processes. Similarly, employing larger TiO_2_ clusters or introducing surface defects would help clarify how real semiconductor environments influence dye anchoring, orbital hybridization, and electron injection. By combining these practical and theoretical directions, the present work can serve as a foundation for guiding the rational design of next-generation sensitizers that deliver strong light harvesting, efficient charge separation, and long-term operational stability.

### 4.9 Conclusion

In a nutshell, we have performed DFT and TD-DFT calculations on various dyes with a D–π–A–A′ framework. These dyes include a thiophene π-bridge, cyanoacrylic acid serving as an electron-acceptor cum anchoring group, and typical electron-donor and electron-acceptor moieties. The electron-donor moieties investigated are coumarin (COU), triphenylamine (TPA), indoline (IN), carbazole (CAR), diphenylamine (DPA), tetrahydroquinoline (THQ), triazatruxene (TAT), azulene (AZ), and julolidine (JUD). Besides, the electron-acceptor moieties used include quinoline (QN), [1,2,5]thiadiazole[3,4-*c*]pyridine (PY), phthalimide (PTM), benzothiadiazole (BTZ), naphthalenediimide (NDI), benzothiazole (BZ), and pyridoquinazolinone (PYQ). The dyes designed are assigned as follows: COU–QN, TPA–PY, IN–PTM, CAR–BTZ, DPA–NDI, THQ–BZ, TAT–BZ, TPA–PYQ, AZ–QN, and JUD–BTZ. Among the dyes designed, the TPA–PYQ dye has the lowest *Δ*_H–L_ value of 1.674 eV, which decreases further to 1.281 eV when bound to the Ti_5_O_10_ cluster. According to the band-alignment plot relative to the conduction band (CB) of TiO_2_ and the redox potential of the I^−^/I_3_^−^ electrolyte, the GSOP values of all the designed dyes are lower than the redox potential of the I^−^/I_3_^−^ couple (*i.e.*, −4.85 eV). On the other hand, nearly all of the dyes show ESOP values above the conduction band of TiO_2_ (*i.e.*, −4.05 eV), with the exception of AZ–QN. The Ti–O bond lengths and negative adsorption energies of the dye–clusters indicate that they all undergo chemisorption onto the TiO_2_ semiconductor surface, suggesting effective electron transfer from the dye's LUMO to the conduction band of TiO_2_. Based on the absorption properties, it can be observed that the *λ*_max_ of all the designed dyes undergo red-shift when complexed with the Ti_5_O_10_ cluster. Among the dye–clusters, JUD–BTZ–Ti_5_O_10_, TPA–PY–Ti_5_O_10_, and TPA–PYQ–Ti_5_O_10_, which have lower *E*_b_ values, show a higher ability for exciton dissociation and efficient charge transfer, leading to improved *J*_sc_ values. Consequently, the dyes' performance is enhanced upon binding to the TiO_2_ surface. In brief, all of our designed dyes are promising candidates for the development of DSSCs. However, among the dyes studied, TPA–PY, TPA–PYQ, and JUD–BTZ stand out as the most promising candidates for DSSCs due to their favorable HOMO–LUMO gaps, optimal absorption properties, and enhanced exciton dissociation and charge transfer when bound to TiO_2_. The present work explores new donor–acceptor combinations and incorporates detailed structure–property analyses, providing deeper insight into how molecular modifications influence electronic and optical behavior. These results not only identify specific high-performing sensitizers but also demonstrate the potential of D–π–A–A′ dyes for rational design strategies aimed at next-generation DSSCs. These findings provide a foundation that can guide future work in optimizing donor–acceptor combinations, extending absorption into the near-infrared region, and improving stability under operational conditions. Overall, the insights offered here can serve as a support for both theoretical and experimental exploration of such dyes for next-generation sensitizers.

## Conflicts of interest

There are no conflicts to declare.

## Supplementary Material

RA-015-D5RA07258A-s001

## Data Availability

The data supporting this article have been included as part of the supplementary information (SI). Supplementary information: coordinates of the designed dyes studied at B3LYP-D3/6-31G(d,p) level of theory in the angstrom unit; optimized structure of test compound (ZXY-3); calculated energies of HOMO, LUMO, *Δ*_H–L_, and *λ*_max_ values of the test compound using different functionals, the dihedral angle values of the designed dyes, calculated values of GSOP, ESOP, Δ*G*^reg^ and Δ*G*^inj^ for the designed dyes, estimated values of IP and EA of the studied dye systems, calculated values of *λ*_+_, *λ*_−_, and *λ*_tot_ for the designed dyes, representative diagram for atomic indices and their grouping in the COU–QN molecule, justification for using Ti_5_O_10_ cluster. See DOI: https://doi.org/10.1039/d5ra07258a.
