# Supplementary material for: Theoretical design and analysis of D–π–A–A′ organic dyes for enhanced efficiency in DSSCs by modifying donor (D) and acceptor (A) moieties
Source: RSC Adv. 2025 Nov 3;15(49):42099–112. doi: 10.1039/d5ra07258a (PMC12580974; doi:10.1039/d5ra07258a)
Supplement: RA-015-D5RA07258A-s001 [file RA-015-D5RA07258A-s001.pdf]

Supplementary Information (SI) for RSC Advances.
This journal is © The Royal Society of Chemistry 2025

Supporting Information

Theoretical Design and Analysis of D-π-A-A′ Organic Dyes for Enhanced Efficiency in DSSCs by Modifying Donor (D) and Acceptor (A) Moieties

Smiti Rani Bora, Banashmita Barman and Dhruba Jyoti Kalita*
Department of Chemistry, Gauhati University, Guwahati-781014, India
E-mail: dhrubajyoti.kalita@gauhati.ac.in

Table S1 : Coordinates of the designed dyes studied at B3LYP-D3/6-31G(d, p) level of theory in the angstrom unit.

1. **COU-QN**

Atoms	Coordinates (Angstroms)		
	X	Y	Z
C	-7.917415	0.104940	-0.822658
C	-9.011636	-0.731027	-0.660257
C	-6.645645	-0.281152	-0.351895
C	-8.858745	-1.973551	-0.023569
C	-6.522411	-1.531789	0.280056
C	-7.615104	-2.380402	0.449109
C	-5.468160	0.523085	-0.478382
C	-4.252471	0.116299	-0.000370
C	-4.153162	-1.211985	0.638972
O	-5.312081	-1.956542	0.747997
O	-3.135066	-1.703908	1.073228
H	-8.024635	1.068139	-1.313450
H	-9.986958	-0.426003	-1.025022
H	-9.716925	-2.626084	0.102014
H	-7.469158	-3.334669	0.942479
H	-5.565917	1.482925	-0.976312
C	-3.049092	0.935600	-0.100662
C	-2.974259	2.241947	-0.554285
C	-1.666804	2.771793	-0.560335
C	-0.713846	1.877395	-0.113181
S	-1.451315	0.361981	0.347290
C	0.729902	2.096209	0.003382
C	1.227726	3.422901	0.200079

C	2.572370	3.680665	0.307123
C	3.515405	2.625246	0.235008
C	3.035288	1.289116	0.044476
C	1.641999	1.054347	-0.072279
H	-3.838888	2.810441	-0.875538
H	-1.429846	3.769899	-0.909513
H	0.518255	4.238790	0.290300
H	2.947270	4.686557	0.462787
H	1.301724	0.037046	-0.243967
C	3.986049	0.248674	-0.031677
C	5.340782	0.545197	0.085050
C	5.694152	1.921536	0.282896
N	4.843777	2.917237	0.354241
C	6.433897	-0.405155	0.028738
C	6.453778	-1.750643	-0.179164
C	5.293618	-2.550382	-0.406470
C	7.787085	-2.426700	-0.172966
O	7.681502	-3.754130	-0.390033
O	8.845914	-1.860408	0.004813
H	8.584498	-4.112056	-0.370038
H	7.425611	0.020218	0.174113
H	3.640830	-0.767843	-0.177861
H	6.749351	2.178155	0.381645
N	4.336776	-3.187279	-0.592347

2. **TPA-PY**

Atoms	Coordinates (Angstroms)		
	X	Y	Z
C	-4.750989	0.201536	0.122409
C	-3.787891	-0.822745	0.043120
C	-4.310947	1.509523	0.405003
C	-2.445246	-0.548400	0.249051
C	-2.965042	1.776833	0.596339
C	-1.993704	0.756823	0.530389
H	-4.103783	-1.836229	-0.175585
H	-1.733024	-1.366511	0.197461
H	-2.658414	2.799219	0.791147
H	-5.034785	2.313913	0.467289
C	-0.584839	1.060128	0.749271

C	1.931567	1.055559	0.817764
C	-0.044361	2.151529	1.420746
C	1.356579	2.147804	1.460050
S	0.683032	0.018147	0.145188
C	3.346984	0.817512	0.712061
C	3.920257	-0.325575	0.038376
C	5.357766	-0.468504	-0.048098
C	6.196821	0.538254	0.534416
C	5.500295	1.551394	1.204246
N	4.168230	1.705516	1.283450
N	3.245400	-1.332802	-0.515969
N	5.740621	-1.597424	-0.645269
S	4.368970	-2.388516	-1.088448
H	-0.653797	2.910307	1.895937
H	1.967756	2.898371	1.944317
H	6.077196	2.326541	1.706334
C	7.632797	0.598572	0.517452
C	8.574284	0.007998	-0.272753
C	8.357637	-0.792855	-1.431910
C	10.021345	0.329087	0.042993
O	10.954563	-0.198520	-0.774527
O	10.343827	1.022198	0.980961
H	10.546446	-0.718911	-1.486455
H	8.071771	1.266839	1.256954
N	8.373706	-1.404025	-2.425438
N	-6.115525	-0.074075	-0.075561
C	-6.667432	-1.325657	0.322210
C	-6.972507	0.896160	-0.670140
C	-7.546725	-2.009108	-0.529596
C	-6.351330	-1.876111	1.572907
C	-8.102772	-3.222655	-0.130188
C	-6.897511	-3.099354	1.955732
C	-7.778468	-3.777048	1.109969
C	-8.236541	1.150177	-0.119103
C	-6.567767	1.592781	-1.818222
C	-9.082011	2.084557	-0.713555
C	-7.412573	2.539049	-2.394551
C	-8.674344	2.787320	-1.849514
H	-8.783165	-3.742604	-0.798089
H	-6.644455	-3.515861	2.926226
H	-8.208580	-4.725976	1.414568
H	-7.790478	-1.580459	-1.495846

H	-5.677873	-1.340989	2.234045
H	-8.547621	0.609215	0.768300
H	-7.088039	3.072565	-3.283047
H	-5.592941	1.387868	-2.248060
H	-9.333223	3.519172	-2.306163
H	-10.059157	2.272167	-0.278388

3. **IN-PTM**

Atoms	Coordinates (Angstroms)		
	X	Y	Z
C	-3.097473	-0.726729	-0.179221
C	-2.525789	-1.902170	-0.638055
C	-1.121467	-1.839702	-0.702014
C	-0.584200	-0.619549	-0.310600
S	-1.883004	0.488084	0.109864
H	-3.096235	-2.788726	-0.889681
H	-0.512338	-2.667890	-1.042782
C	0.846347	-0.354292	-0.180714
C	1.507761	0.890859	-0.235806
C	2.903468	0.993163	-0.134144
C	1.707823	-1.468198	0.022647
C	3.081628	-1.364920	0.122142
C	3.747421	-0.116108	0.040153
C	0.965466	2.271377	-0.442447
C	3.283698	2.441181	-0.273230
N	2.080190	3.112462	-0.459163
O	-0.179817	2.653677	-0.569320
O	4.379665	2.964235	-0.239992
C	5.185566	0.072783	0.136515
C	6.192764	-0.838528	0.241884
C	6.028649	-2.256955	0.271192
C	7.590203	-0.304866	0.325790
O	8.502410	-1.296875	0.413601
O	7.880952	0.871708	0.317261
H	9.373011	-0.868519	0.463548
H	5.522303	1.105604	0.114013
H	1.266878	-2.450571	0.139838
H	3.649616	-2.270445	0.286231
N	5.890131	-3.412968	0.294450
H	2.012617	4.114027	-0.570144

C	-4.497060	-0.438434	0.021805
C	-6.521094	0.381494	0.637639
C	-6.726204	-0.747356	-0.207017
C	-9.088805	-0.496466	-0.044600
H	-10.095003	-0.816391	-0.297734
C	-8.910349	0.618298	0.803410
H	-9.784085	1.135274	1.188213
C	-7.642088	1.061009	1.149445
H	-7.510905	1.920256	1.800351
C	-8.003029	-1.194597	-0.559628
H	-8.140733	-2.054572	-1.208331
C	-5.109100	0.554273	0.764133
H	-4.595215	1.298622	1.355559
N	-5.484347	-1.237953	-0.547874
H	-5.314162	-1.932167	-1.256884

4. **CAR-BTZ**

Atoms	Coordinates (Angstroms)		
	X	Y	Z
N	6.952283	0.024771	0.396671
C	6.238010	1.207172	0.530184
C	4.856741	0.947074	0.308621
C	6.710190	2.483523	0.850552
C	5.776061	3.506320	0.984655
C	4.403599	3.264311	0.805231
C	3.939546	1.996675	0.468809
C	8.385086	-0.118164	0.539463
C	6.071027	-0.994776	0.073820
C	4.750466	-0.467003	-0.007593
C	6.350545	-2.349535	-0.123261
C	3.686030	-1.342601	-0.318224
C	5.279787	-3.198151	-0.387578
C	3.972673	-2.706528	-0.483095
H	8.760521	0.633955	1.236241
H	8.907893	-0.005799	-0.418174
H	8.618771	-1.101198	0.955079
H	7.768408	2.676507	0.991523
H	6.116404	4.506855	1.234314
H	3.695878	4.078082	0.928776
H	3.163647	-3.385135	-0.732956

H	5.461960	-4.257076	-0.542312
H	7.364148	-2.732085	-0.072520
H	2.879095	1.821492	0.339526
C	2.311579	-0.862095	-0.519465
C	1.870816	0.170816	-1.318492
C	0.465042	0.324889	-1.331095
C	-0.204240	-0.596031	-0.542099
S	0.959252	-1.664526	0.240185
C	-1.630027	-0.750230	-0.316207
C	-2.601709	0.204425	-0.803156
C	-4.022181	0.039595	-0.528502
C	-4.506969	-1.077002	0.233747
C	-2.165046	-1.834843	0.370983
C	-3.544857	-1.999620	0.616765
N	-2.336571	1.283817	-1.545329
N	-4.783584	0.979618	-1.091158
S	-3.773938	2.003517	-1.880040
H	2.552008	0.786490	-1.893053
H	-0.057997	1.075547	-1.905937
H	-1.499165	-2.609567	0.736921
H	-3.861152	-2.887281	1.157639
C	-5.905383	-1.330691	0.535163
C	-6.928278	-0.462081	0.756562
C	-8.252200	-0.990676	0.894188
C	-6.838241	1.024251	0.922755
O	-7.704223	1.799527	0.589758
O	-5.713293	1.393434	1.577374
N	-9.317736	-1.442577	1.013947
H	-6.169801	-2.386081	0.573601
H	-5.734665	2.364215	1.616666

5. **DPA-NDI**

Atoms	Coordinates (Angstroms) X	Y	Z
C	-11.287906	-1.387122	0.957009
C	-12.483639	-0.886472	0.438859
C	-10.099619	-0.669618	0.833979
C	-12.479296	0.349997	-0.209876
C	-10.089122	0.562436	0.159051

C	-11.293990	1.065650	-0.358283
N	-8.925762	1.339818	0.035122
C	-7.606715	0.924971	-0.097721
C	-7.245303	-0.374356	-0.505819
C	-5.909618	-0.722997	-0.639455
C	-4.873186	0.199953	-0.399588
C	-5.245660	1.502114	-0.012732
C	-6.576040	1.855480	0.142725
H	-11.278765	-2.338560	1.480850
H	-13.405991	-1.448601	0.543827
H	-13.401141	0.756769	-0.615151
H	-11.291720	2.016842	-0.884544
H	-9.073147	2.337427	-0.000728
H	-6.831086	2.865745	0.452417
H	-4.480557	2.253737	0.158387
H	-5.663017	-1.739527	-0.928605
H	-8.013456	-1.104876	-0.727730
H	-9.186606	-1.048050	1.279135
C	-3.477257	-0.193543	-0.559851
C	-2.957820	-1.218614	-1.332588
C	-1.560424	-1.363879	-1.227525
C	-0.961710	-0.446675	-0.386494
S	-2.189690	0.612979	0.304422
H	-3.572874	-1.831312	-1.980424
H	-0.995497	-2.111262	-1.765957
C	0.454407	-0.186740	-0.134983
C	1.473238	-1.155202	0.031690
C	2.831962	-0.735056	0.149879
C	0.837683	1.187828	-0.101834
C	3.184406	0.645214	0.124164
C	2.141666	1.598656	0.015812
C	1.180362	-2.610217	0.192656
N	2.269446	-3.487831	0.351901
O	0.055570	-3.087133	0.214821
C	1.922704	-4.908166	0.496839
C	3.614665	-3.124535	0.399453
C	3.886987	-1.669666	0.299740
C	5.201585	-1.252432	0.391317
C	5.556922	0.113811	0.353704
C	4.531181	1.063197	0.220951
C	2.439237	3.052959	0.003765
C	4.859802	2.512191	0.123652

N	3.789257	3.413246	0.076519
C	4.077266	4.853740	0.027737
H	1.356327	-5.237600	-0.376048
H	2.848391	-5.469747	0.588958
H	1.297420	-5.045279	1.380936
H	3.779433	5.257797	-0.942409
H	3.499366	5.358839	0.802607
H	5.143484	4.993319	0.182869
O	1.572067	3.910134	-0.071665
O	6.017043	2.912943	0.088995
O	4.507722	-3.949275	0.531899
H	5.961723	-2.013138	0.524690
H	0.082989	1.957379	-0.217596
C	6.965787	0.485752	0.534267
C	8.020628	-0.093114	-0.085002
C	7.895362	-1.067528	-1.123671
C	9.415407	0.349448	0.302540
O	9.614782	1.142062	1.190424
O	10.437074	-0.190971	-0.392673
N	7.899972	-1.855262	-1.982561
H	7.210493	1.270621	1.241557
H	10.121100	-0.804871	-1.075298

6. **THQ-BZ**

Atoms	Coordinates (Angstroms)		
	X	Y	Z
C	7.337625	-0.627162	0.799548
C	8.579482	0.073583	0.232493
C	6.057527	-0.018173	0.260050
C	8.439575	1.588949	0.389554
C	6.062065	1.311016	-0.230788
N	7.236992	2.036092	-0.300004
H	7.134340	3.032389	-0.425466
C	4.865240	-0.730834	0.263894
C	3.651027	-0.183893	-0.192442
C	3.676374	1.141359	-0.668002
C	4.852915	1.870501	-0.689007
H	8.411990	1.845192	1.462158
H	9.484092	-0.269683	0.744011

H	7.366554	-1.698741	0.573034
H	4.873571	-1.740589	0.665873
H	4.851284	2.886094	-1.076800
H	8.688732	-0.162425	-0.832181
H	7.347478	-0.544549	1.895577
H	9.299734	2.105056	-0.048827
C	2.426366	-0.976559	-0.168199
C	2.301095	-2.356825	-0.196932
C	0.964988	-2.806033	-0.157689
C	0.041963	-1.778237	-0.098602
C	-1.391531	-1.843823	-0.056627
N	-2.169553	-0.793423	-0.020052
S	-2.250298	-3.413099	-0.047124
S	0.847894	-0.221407	-0.073884
C	-3.765271	-2.529941	0.008219
C	-3.498476	-1.138942	0.018724
C	-5.067124	-3.021577	0.038346
C	-6.116617	-2.099932	0.079006
C	-5.879213	-0.724089	0.089542
C	-4.571914	-0.205128	0.061188
H	3.152952	-3.020330	-0.280277
H	0.682407	-3.852735	-0.188604
H	-5.264625	-4.088330	0.030875
H	-7.139864	-2.459927	0.104046
H	-6.724685	-0.050388	0.124273
H	2.767959	1.597557	-1.050318
C	-4.208763	1.199734	0.067383
C	-4.959726	2.335753	0.059501
C	-4.328524	3.702084	0.124114
C	-6.388829	2.354206	0.081446
N	-7.552632	2.359050	0.082917
O	-3.049290	3.808422	-0.321731
O	-4.907577	4.664750	0.562591
H	-2.792732	3.020525	-0.821394
H	-3.130955	1.339360	0.104922

7. **TAT-BZ**

Atoms	Coordinates (Angstroms)		
	X	Y	Z
C	-8.087395	-1.748846	0.835097

C	-9.463568	-1.619656	1.006914
C	-10.100911	-0.381559	0.819785
C	-7.333309	-0.625850	0.470844
C	-9.377349	0.753374	0.457164
C	-8.000547	0.617320	0.286823
C	-5.933395	-0.380782	0.210571
C	-5.803330	0.991642	-0.119567
C	-4.763098	-1.161400	0.216348
C	-3.490472	-0.623815	-0.093194
C	-3.416706	0.746340	-0.413101
C	-4.562163	1.578847	-0.433790
N	-7.055598	1.569680	-0.067136
H	-7.253226	2.537126	-0.257965
H	-11.174827	-0.305710	0.959486
H	-9.868704	1.710885	0.312045
H	-10.052917	-2.486505	1.289280
N	-2.316699	1.511186	-0.746113
C	-4.117437	2.904661	-0.797720
C	-2.709308	2.820645	-0.983690
C	-4.749658	4.140961	-0.984422
C	-3.986916	5.249064	-1.344791
C	-2.597130	5.141939	-1.522977
C	-1.940027	3.925770	-1.344721
H	-4.471393	6.209480	-1.490777
H	-2.024216	6.019956	-1.804725
H	-0.866288	3.840826	-1.483675
H	-5.822921	4.246061	-0.852443
H	-1.370583	1.174457	-0.800863
N	-4.608302	-2.507773	0.501575
C	-3.277312	-2.861542	0.388356
C	-2.537467	-1.702480	0.015546
C	-2.658114	-4.096398	0.581133
C	-1.282404	-4.173600	0.400362
C	-0.516688	-3.043953	0.021992
C	-1.159186	-1.809837	-0.169385
C	0.929045	-3.178686	-0.165455
C	1.639095	-4.301461	-0.552293
C	3.031540	-4.085074	-0.646793
C	3.406366	-2.792695	-0.333018
S	2.011093	-1.829250	0.106530
H	-3.228887	-4.971199	0.877296
H	-0.777670	-5.116489	0.580862

H	-0.576483	-0.951835	-0.489979
H	-5.354942	-3.133434	0.752328
H	1.160424	-5.242041	-0.795742
H	3.739007	-4.848446	-0.951655
H	-7.614803	-2.715750	0.984707
C	4.715530	-2.197630	-0.327147
N	4.943190	-0.947495	-0.025735
S	6.170915	-3.148856	-0.742810
C	7.129402	-1.704129	-0.476939
C	6.279228	-0.635625	-0.098064
C	8.506326	-1.535454	-0.592519
C	9.036880	-0.271940	-0.322553
C	8.220165	0.796694	0.052181
C	6.825695	0.650806	0.175731
C	5.890453	1.690854	0.562450
C	6.082879	3.006363	0.865328
C	7.350915	3.668496	0.852745
C	4.947514	3.891605	1.248409
O	5.064584	5.065456	1.530809
O	3.750105	3.252912	1.253677
N	8.389955	4.192393	0.836935
H	9.150497	-2.358715	-0.882911
H	10.107446	-0.115589	-0.404908
H	8.679709	1.754967	0.251786
H	4.861324	1.350990	0.616347
H	3.099133	3.919974	1.525566

8. **TPA-PYQ**

Atoms	Coordinates (Angstroms) X	Y	Z
C	6.416118	0.045183	0.009344
C	5.379206	-0.594481	0.710243
C	6.103669	1.186559	-0.750764
C	4.080066	-0.109931	0.648887
C	4.803873	1.668728	-0.800817
C	3.757655	1.035591	-0.102393
H	5.602342	-1.469498	1.310334
H	3.307004	-0.611093	1.224144
H	4.585760	2.530086	-1.424231

H	6.887272	1.683246	-1.312098
C	2.396891	1.567252	-0.166108
C	-0.116985	1.881398	-0.159859
C	1.996146	2.864265	-0.412718
C	0.591012	3.039600	-0.408714
S	0.994110	0.541610	0.069848
H	2.699099	3.674122	-0.567706
H	0.112460	3.992558	-0.601319
N	7.738324	-0.448022	0.063387
C	7.980635	-1.848432	0.120765
C	8.840654	0.450067	0.059556
C	8.955197	-2.361359	0.989956
C	7.255436	-2.730720	-0.694525
C	9.202060	-3.731779	1.033940
C	7.495652	-4.101466	-0.628837
C	8.472037	-4.610352	0.230490
C	9.974034	0.174569	-0.720364
C	8.812347	1.615755	0.840408
C	11.059098	1.048260	-0.711385
C	9.894448	2.493106	0.827561
C	11.025232	2.214151	0.056758
H	9.960180	-4.114850	1.710842
H	6.925762	-4.772813	-1.264575
H	8.661757	-5.678322	0.273100
H	9.514558	-1.679525	1.621561
H	6.505771	-2.334265	-1.370924
H	9.996321	-0.726178	-1.324385
H	9.858228	3.391898	1.436342
H	7.939526	1.825960	1.449285
H	11.869699	2.896245	0.055663
H	11.930020	0.821751	-1.319574
C	-1.564723	1.703284	-0.085206
C	-3.561776	0.313816	-0.168157
C	-4.399267	1.438562	0.057569
C	-4.151055	-1.003980	-0.338809
C	-6.317523	0.175711	-0.014525
C	-8.389227	-1.145984	-0.087495
C	-10.851366	-0.461511	0.173768
C	-10.696582	0.942056	0.378517
C	-12.240293	-1.022885	0.175457
N	-5.586175	-1.007365	-0.247690
N	-5.758799	1.355451	0.133408

O	-3.548859	-2.048548	-0.541930
O	-12.506545	-2.195335	0.012033
N	-10.564631	2.086660	0.545168
O	-13.169078	-0.068181	0.380937
H	-14.034117	-0.510565	0.367606
C	-2.169131	0.455721	-0.240921
H	-1.583803	-0.436383	-0.438115
C	-2.410335	2.826325	0.151290
H	-1.960848	3.801642	0.305568
C	-3.778999	2.702993	0.215112
H	-4.416219	3.561001	0.400054
C	-6.233675	-2.224414	-0.396314
H	-5.566577	-3.057555	-0.570091
C	-7.579964	-2.319436	-0.323654
H	-8.048817	-3.289042	-0.445377
C	-7.740057	0.060747	0.060089
H	-8.259932	0.991352	0.240180
C	-9.828962	-1.337444	-0.025023
H	-10.165665	-2.363254	-0.160809

9. **AZ-QN**

Atoms	Coordinates (Angstroms)		
	X	Y	Z
C	-3.263103	0.232604	0.412083
C	-3.312918	1.534325	0.872111
C	-2.064872	2.204823	0.826552
C	-1.030879	1.431777	0.342297
S	-1.628685	-0.158834	-0.097026
C	0.377036	1.798112	0.193100
C	0.736620	3.178016	0.067020
C	2.044487	3.575736	-0.061504
C	3.089741	2.618732	-0.087119
C	2.749048	1.231776	0.026040
C	1.391085	0.851887	0.169315
H	-4.213434	1.971013	1.286233
H	-1.921760	3.215868	1.190096
H	-0.051240	3.923720	0.051654
H	2.312006	4.622289	-0.160676
H	1.160499	-0.203852	0.281572
C	3.799252	0.289493	0.001409

C	5.113775	0.726918	-0.133951
C	5.324381	2.141684	-0.243085
N	4.377887	3.049455	-0.222578
C	6.294599	-0.111939	-0.175528
C	6.449797	-1.462798	-0.096166
C	5.377318	-2.392028	0.057188
C	7.842373	-1.999290	-0.175306
O	7.871732	-3.345544	-0.085372
O	8.837868	-1.316787	-0.304771
H	8.805395	-3.607473	-0.145088
H	7.237426	0.420552	-0.290054
H	3.561128	-0.763794	0.088744
H	6.345426	2.508751	-0.351477
N	4.490306	-3.135932	0.182588
C	-4.315708	-0.762099	0.325451
C	-5.679130	-0.541625	-0.011597
C	-6.360717	-1.868770	0.077728
C	-7.571992	0.937589	-0.772704
H	-7.770870	1.962866	-1.074545
C	-6.244195	0.657731	-0.432291
H	-5.553924	1.493582	-0.516196
C	-8.676854	0.081496	-0.773550
H	-9.621008	0.544166	-1.054971
C	-8.745894	-1.284267	-0.485405
H	-9.730551	-1.737038	-0.569746
C	-7.708160	-2.149391	-0.126430
H	-7.988806	-3.192330	0.015489
C	-5.374911	-2.811199	0.427716
H	-5.551179	-3.868477	0.578709
C	-4.156244	-2.148416	0.571047
H	-3.217910	-2.609771	0.857237

10. **JUD-BTZ**

Atoms	Coordinates (Angstroms)		
	X	Y	Z
C	2.031709	0.762407	-0.104214
C	1.533998	2.042332	-0.292124
C	0.128275	2.119303	-0.295645
C	-0.504149	0.896935	-0.113489
S	0.710013	-0.370294	0.086677

C	-1.913799	0.590547	-0.077643
C	-2.935975	1.614366	-0.131110
C	-4.346430	1.262518	-0.084996
C	-4.783592	-0.108491	0.014842
C	-2.396853	-0.716997	0.016467
C	-3.760368	-1.055055	0.061691
N	-2.727331	2.930993	-0.220230
N	-5.159391	2.319602	-0.141903
S	-4.203663	3.651130	-0.244793
H	-1.686982	-1.536974	0.052179
H	-4.009157	-2.105857	0.134391
H	2.175754	2.899348	-0.456295
H	-0.429001	3.033824	-0.440339
C	-6.197998	-0.368320	0.056072
C	-6.900152	-1.538892	0.146258
C	-6.309427	-2.834560	0.222125
C	-8.386873	-1.444686	0.166086
O	-9.019294	-0.409134	0.109299
O	-8.972456	-2.661072	0.256111
N	-5.809235	-3.885139	0.283119
H	-6.827423	0.517092	0.007965
H	-9.930568	-2.501867	0.262553
C	3.421942	0.334465	-0.041415
C	6.154153	-0.456731	0.069174
C	5.137691	-1.403613	-0.228445
N	7.493934	-0.830999	0.069566
C	6.823997	1.918532	0.708563
H	6.857568	2.019706	1.802669
H	6.539308	2.901512	0.317953
C	5.777991	0.888952	0.330238
C	4.441162	1.253472	0.267137
H	4.180387	2.283455	0.493735
C	3.812804	-0.993654	-0.284235
H	3.060561	-1.733581	-0.546765
C	8.500340	0.074624	0.610304
H	8.548453	0.000063	1.711191
H	9.476028	-0.241774	0.225746
C	8.209085	1.515989	0.201111
H	8.245608	1.588401	-0.891953
H	8.981761	2.178375	0.604213
C	7.849581	-2.244636	0.046434
H	7.785936	-2.685528	1.057067

H	8.895447	-2.321130	-0.270090
C	6.945512	-3.013660	-0.913158
H	7.237227	-4.068523	-0.931899
H	7.081034	-2.612821	-1.924273
C	5.488517	-2.857180	-0.476123
H	5.325953	-3.432238	0.446517
H	4.811817	-3.282211	-1.225271

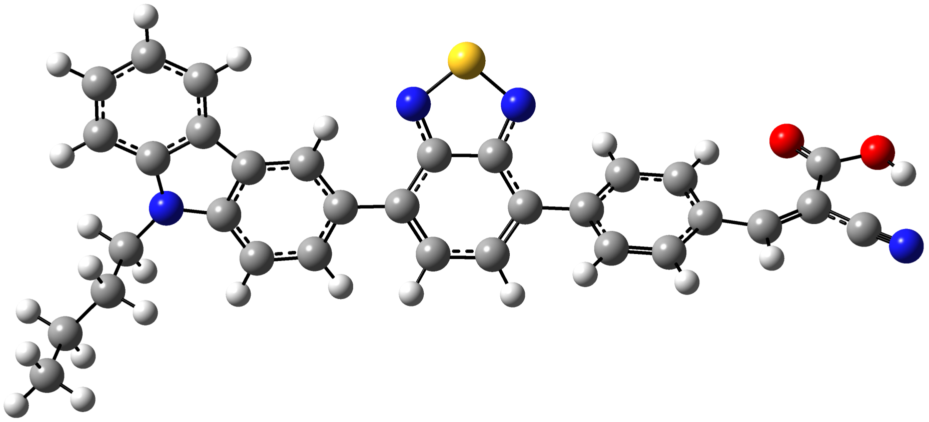

Fig. S1 : Optimized structure of test compound (ZXY-3).

Table S2 : Calculated energies of HOMO, LUMO, Δ_{H-L}, and λ_{max} values of the test compound using different functionals .

**Functional**	**HOMO (eV)**	**LUMO (eV)**	**Δ_{H-L} (eV)**	**λ_{max} (nm)**
Experimental	-5.527	-3.060	2.467	391
B3LYP	-5.423	-2.872	2.551	551
B3LYP-D3	-5.420	-2.862	2.558	550
CAM-B3LYP	-6.623	-1.723	4.900	413
B3PW91	-5.540	-2.975	2.565	549
HSEH1PBE	-5.306	-3.144	2.162	560
WB97XD	-7.212	-1.187	6.025	401

Table S3 : The dihedral angle values of the designed dyes.

Dyes	D-π (º)	π-A (º)	A-A (º)
COU-QN	6.64	26.87	2.16
TPA-PY	21.67	0.61	9.65
IN-PTM	-16.21	-22.96	4.16
CAR-BTZ	48.89	-8.69	-33.08
DPA-NDI	23.58	-42.44	46.73
THQ-BZ	-24.96	1.13	-2.27
TAT-BZ	21.73	-5.93	10.71
TPA-PYQ	-25.27	-20.17	0.23
AZ-QN	36.60	-23.68	-0.23
JUD-BTZ	21.25	-5.17	-0.03

Table S4 : Calculated values of GSOP, ESOP, ΔG^{reg} and ΔG^{inj} for the designed dyes.

Dyes	GSOP (eV)	ESOP (eV)	ΔG^{reg} (eV)	ΔG^{inj} (eV)
COU-QN	-6.741	-2.958	1.941	1.042
TPA-PY	-6.243	-3.859	1.443	0.141
IN-PTM	-6.612	-3.625	1.812	0.375
CAR-BTZ	-6.599	-3.826	1.799	0.174
DPA-NDI	-6.321	-4.005	1.521	-0.005
THQ-BZ	-6.327	-3.228	1.527	0.772
TAT-BZ	-6.065	-3.268	1.265	0.732
TPA-PYQ	-6.057	-3.502	1.257	0.498
AZ-QN	-6.389	-4.130	1.589	-0.130
JUD-BTZ	-6.155	-3.776	1.355	0.224

Table S5: Estimated values of IP and EA of the studied dye systems.

**Dyes**	**IP (eV)**	**EA (eV)**
COU-QN	6.74	1.73
TPA-PY	6.24	2.34
IN-PTM	6.61	1.95
CAR-BTZ	6.59	2.01
DPA-NDI	6.38	2.65
THQ-BZ	6.32	1.55
TAT-BZ	6.25	1.42
TPA-PYQ	5.96	1.94
AZ-QN	6.38	1.49
JUD-BTZ	6.15	1.79

Table S6: Calculated values of λ_+, λ_-, and λ_{tot} for the designed dyes.

**Dyes**	**λ_+ (eV)**	**λ_- (eV)**	**λ_{tot} (eV)**
COU-QN	0.134	0.092	0.226
TPA-PY	0.619	0.168	0.787
IN-PTM	0.131	0.183	0.314
CAR-BTZ	0.087	0.434	0.521
DPA-NDI	0.381	0.187	0.568
THQ-BZ	0.132	0.131	0.263
TAT-BZ	0.070	0.147	0.217
TPA-PYQ	0.126	0.269	0.395
AZ-QN	0.168	0.628	0.796
JUD-BTZ	0.156	0.135	0.291

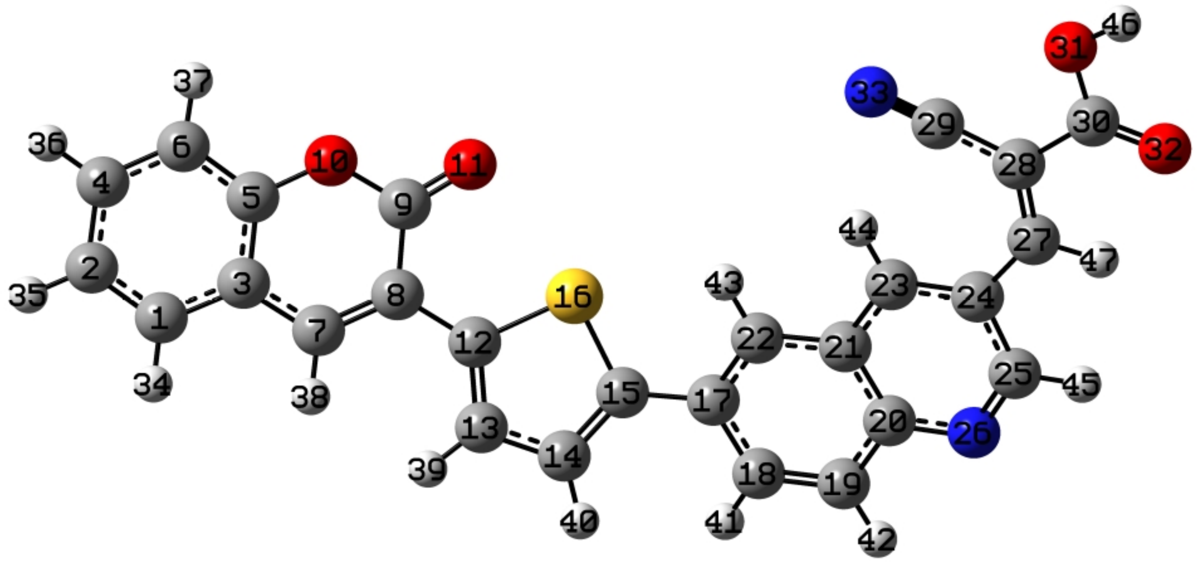

Fig S2: Representative diagram for Atomic indices and their grouping in the COU-QN molecule. Hydrogen atoms are listed last. Atoms 1–11 correspond to the donor (D), 12–16 to the π-spacer, 17–26 to the acceptor (A), and 27–31 to the anchoring group (A′).

Justification for Using a $(TiO_2)_5$ Cluster

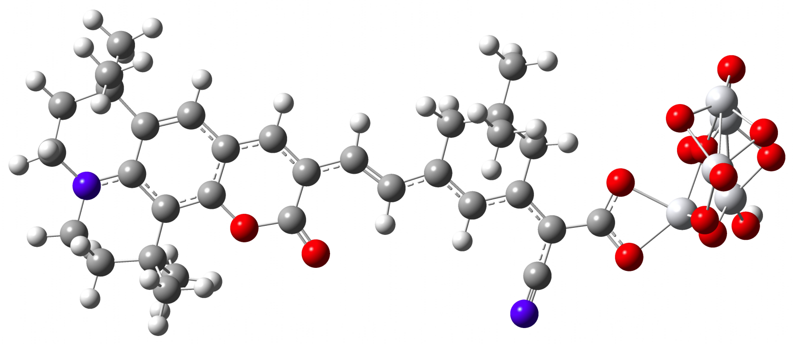

Fig. S3 : Optimized structure of the NKX-2753-Ti_5O_{10} cluster.

Dye-Cluster	Δ_{H-L} (eV)
NKX-2753-Ti_9O_{18}	1.319
NKX-2753-Ti_5O_{10}	1.317

Table 1: Comparison of Δ_{H-L} values for dye-clusters.

To support our decision to use a $(TiO_2)_5$ cluster, we have referenced a study that utilized larger $(TiO_2)_9$ clusters (DOI: 510.1039/c1cp22058f3). We then took one of the dyes from that study, NKX-2753, and anchored it to our $(TiO_2)_5$ cluster. After optimizing this system using the same theoretical methods as the original study, we found only a tiny 0.002 eV difference in the calculated Δ_{H-L} values. This negligible difference suggests that increasing the size of the TiO_2 cluster has a minimal impact on the predicted energy gap. Therefore, we believe the **$(TiO_2)_5$ cluster** is a reliable and computationally efficient model for our research.

……………………………………………………………………………………………..
